# Barriers to high school and university students’ physical activity: A systematic review

**DOI:** 10.1371/journal.pone.0265913

**Published:** 2022-04-04

**Authors:** Regina Márcia Ferreira Silva, Carolina Rodrigues Mendonça, Vinicius Diniz Azevedo, Aamir Raoof Memon, Priscilla Rayanne E. Silva Noll, Matias Noll

**Affiliations:** 1 Federal Institute Goiano, Ceres, Goiás, Brazil; 2 Federal University of Goiás, Goiânia, Goiás, Brazil; 3 Institute of Physiotherapy and Rehabilitation Sciences, Peoples University of Medical and Health Sciences for Women, Nawabshah (Shaheed Benazirabad), Pakistan; 4 Faculdade de Medicina USP, São Paulo, Brazil; 5 Department of Sports Science and Clinical Biomechanics, University of Southern Denmark, Odense, Denmark; La Inmaculada Teacher Training Centre (University of Granada), SPAIN

## Abstract

Physical inactivity commonly occurs throughout one’s life, particularly during adolescence and young adulthood. Multiple factors can negatively influence participation in physical activity, but there has been no review examining the barriers to physical activity among high school and university students. Therefore, the aim of this systematic review was to summarize evidence of barriers to the practice of physical activity among high school and university students. The literature search was conducted without time limits using five databases, including CINAHL, Cochrane Library, Embase, PubMed, and Scopus. In total, 59 studies (37 with high school students [n = 22,908] and 22 with university students [n = 15,411]) were included. The main barriers identified in high school and university students were lack of time, lack of motivation, and lack of accessible places. These findings may be useful in designing and implementing evidence-informed interventions and programs for physical activity promotion in students.

## 1. Introduction

Chronic non-communicable diseases (e.g., cancer, diabetes, respiratory, and cardiovascular diseases) are a major current public health issue and responsible for more than 70% of worldwide mortality in adults [1, 2]. In adults, these diseases result in days of lost work and reduced productivity, in addition to affecting quality of life [3]. In children and adolescents, these diseases affect several domains (e.g., social, emotional, cognitive, physical) of wellness, which in turn creates the risk of decline in academic performance and school attendance [4]. Therefore, regular physical activity has been considered a significant factor in the prevention of chronic non-communicable diseases [5–7]. Recent studies have identified physical and psychological benefits associated with regular participation in physical activity. For example, physical benefits resulting from physical activity include body weight regulation [8, 9], blood pressure reduction [10], better bone health [11], and improved muscle strength and function [12]. Furthermore, psychological benefits of physical activity include reduced risk of dementia [13, 14]; reduction of depressive symptoms in youth [15]; improved cognition, brain function, and academic performance [16]; better mental health [17]; and development and preservation of cognitive health throughout life [18]. Regular participation in physical activity is, therefore, essential to maintaining and improving physical and psychological health across the lifespan.

Physical inactivity is described as the “inability to meet specific physical activity guidelines (e.g., 150–300 minutes of moderate intensity or 75–150 minutes of vigorous intensity physical activity per week)” [19–23]. The worldwide prevalence of physical inactivity among adults ranges from 12.3% to 43.7% [24]. Despite the well-documented health benefits of physical activity, most young people (10–24 years old as defined by the World Health Organization) [25] do not meet the physical activity recommendations; that is, more than 81% of adolescents in the world are considered physically inactive [26]. It has been shown that the participation in physical activity tends to decrease with age, and this decline starts in early adolescence [27, 28], with a more pronounced decline during late adolescence and early adulthood [29, 30]. Therefore, measures that can contribute to improved physical activity participation by both adolescents and young adults are encouraged.

Life events and transitions have been shown to have a negative effect on physical activity and other lifestyle behaviors. The transition of leaving school, therefore, is an important time to support individuals to prevent decline in physical activity [31]. Students (adolescents and young adults who attend school, college, or university), whatever the study level, constitute a group that is vulnerable to different lifestyle and behavioral changes [28, 31–34]. Evidence has shown that health behaviors adopted during late adolescence and early adulthood may continue later in life [35]. Individuals in late adolescence are at potential risk of considerable mental health deficits, which if not addressed, may continue to persist and increase in severity in early adulthood. Therefore, regular physical activity may serve as a protective factor against these mental health problems and improve cognitive function [36]. University is a very competitive environment in which students undergo physical and mental changes [37]. Some researchers have reported that starting college and university, particularly the first year, is associated with weight gain, unhealthy eating, sleep problems, and lack of physical activity [38–40]. In addition, previous reviews and large-scale studies have shown that the prevalence of physical inactivity is high in both school and university students [26, 41–45].

Barriers to the practice of physical activity can be broadly categorized into individual, behavioral, and environmental factors [46–49], which can be further grouped into six categories (dimensions): 1) socioeconomic and demographic factors; 2) psychological, emotional, and cognitive factors; 3) sociocultural factors; 4) environmental factors; 5) physical activity characteristics; and 6) behavioral attributes [50–53]. Multiple factors influence physical activity behavior, so the examination of such factors is important, particularly in individuals in late adolescence and early adulthood [54, 55]. As far as we know, only one systematic review from 2014 [56] and an updated systematic review [57] have been published on barriers to physical activity in adolescents. However, these reviews are limited to only studies covering a specific age group (adolescents between 13 and 18 years old) [56, 57], which excludes undergraduate university students. Therefore, there is a need for further research focusing on diverse populations (e.g., children, adolescents, university students) and study designs to advance the knowledge in this area [57, 58].

Although some reviews [59, 60] have examined the determinants of physical activity in relation to a specific category of factors (i.e., psychological, environmental), they are limited in scope. Understanding what factors affect physical activity is important as some have been linked to the success of programs and interventions aimed at improving physical activity and health [61]. Thus, this systematic review aimed to identify barriers to the practice of physical activity among high school, college, and university students. The current systematic review includes different types of studies and covers a broad population group (ranging from high school students who are in their late adolescence to undergraduate students who have just transitioned into young adulthood) and study designs (both qualitative and quantitative). The information obtained from this review can provide a better understanding of the barriers encountered by students in meeting the recommended levels of physical activity, which may be helpful for designing and implementing evidence-informed interventions and programs for physical activity promotion as well as for informing environmental modifications to improve students’ physical activity.

## 2. Methods

### 2.1 Protocol and registration

This systematic review follows the PRISMA guidelines [62] for identification, screening, eligibility, and inclusion of primary studies. The protocol for this review was recently published [58], and it was registered in the PROSPERO (CRD42020198899). Ethical approval was not required because this study does not involve any human participants.

### 2.2 Identification and selection of studies

The literature search was performed on November 5, 2021, using the following five bibliographic databases: CINAHL, Cochrane Library, Embase, PubMed, and Scopus. The search terms for the key concepts—"students," "high school/university," "barriers," and "physical activity"—were combined using Boolean operators (AND/OR), with no restriction on publication year. The search strategy was adapted for each database. The detailed search strategy is described in S1 Table. Secondary searches were performed by manually searching the reference lists of articles included in this review (reference lists of studies eligible for inclusion were searched to find potentially eligible studies).

The eligibility criteria were specified according to the Population, Exposure, Outcomes, and Study (PEOS) framework for the research question [63–65]: "P" referred to high school and/or university students, comprising adolescents or adults of both sexes aged between 10–30 years; "E" corresponded to barriers to physical activity; "O" constituted the practice of physical activity; and "S" referred to studies with qualitative and quantitative designs published during any year in peer-reviewed journals in English, Spanish, or Portuguese.

For this review, studies that targeted students in the aforementioned age group were eligible for inclusion. The World Health Organization defines “adolescents” as individuals aged 10–19 years and “youth” as individuals aged 15–24 years; thus, “young people” are individuals who range in age from 10 to 24 years [25]. The extension of the age range to 30 years was justified by the fact that this age range would also cover university students who are enrolled in undergraduate courses [66–68]. Therefore, the age up to 30 years was meant to cover undergraduate university students.

Physical activity is defined as “any bodily movement produced by skeletal muscles that requires energy expenditure” [69]. Physical activity broadly includes walking, cycling, swimming, playing sports, and performing recreational activities [7]. Barriers refer to factors that prevent or hinder an individual’s participation in physical activity [46].

Systematic or narrative reviews; case studies; opinion articles; letters; replies; conference abstracts; theses or dissertations; book chapters; and studies that included people with physical and/or mental disabilities, groups with chronic diseases, and pregnant or lactating women were excluded. In addition, studies on specific and/or traditional communities (e.g., rural, indigenous, refugees, isolated, and aboriginal) and studies with mixed age samples were excluded.

**Fig 1:**
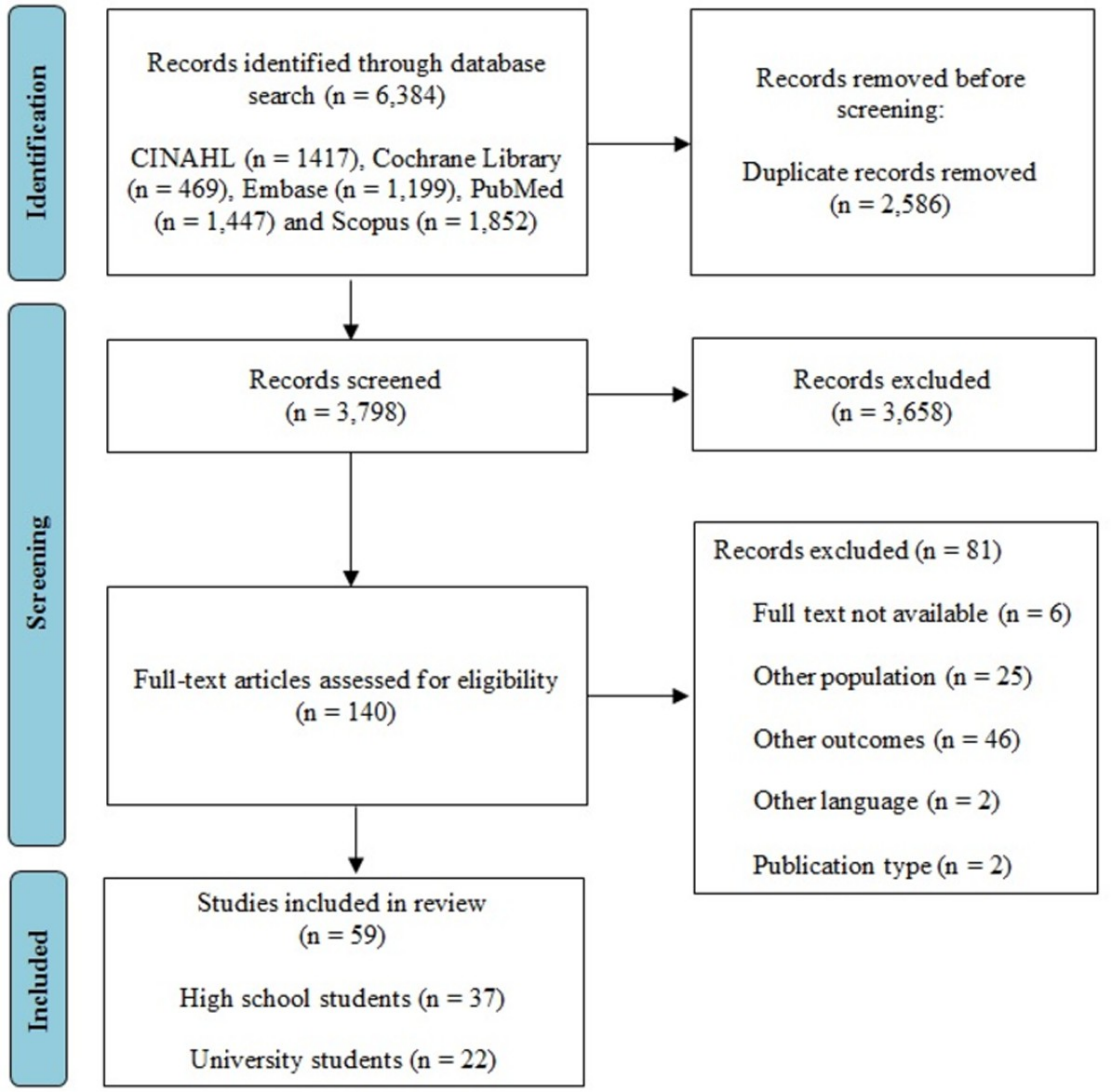
Preferred reporting items for systematic reviews and meta-analyses flow diagram for study selection.

The results of the database searches were imported into the Mendeley software, where duplicate studies were identified and excluded. Two reviewers (RMFS and CRM), who were trained to screen articles, independently evaluated the titles and abstracts of the studies according to the eligibility criteria. After this stage, studies available online was assessed to determine their inclusion. Any disagreements were resolved by involving a third reviewer (MN). All the steps involving study screening were performed in the *Rayyan* [70] software. **Fig 1** shows the selection process of studies included in the current systematic review.

### 2.3. Data extraction

The following data were extracted from the included studies: author and year of publication, type of study, country, population, sex, age group, data collection instrument, and barriers to physical activity. We categorized the results into two groups: (a) high school students and (b) university students. The information was extracted independently by two reviewers (RMFS and CRM), and disagreements were resolved by a third reviewer (MN).

The factors included in the socioeconomic and demographic category were: age, sex, socioeconomic status, anthropometric characteristics, and ethnicity. The psychological, emotional, and cognitive category included: motivation for or interest in physical activity, benefits of physical activity, desire to exercise, mood disorders, perception of health and physical competence, lack of time, lack of desire, and laziness. The factors in the sociocultural category constituted: social support from family, friends/peers, and teachers or significant others. The environmental category included: access to equipment, climate, and program costs. The factors in the physical activity characteristics category were: intensity and subjective feeling of physical effort. Finally, the behavioral attributes category included: history of previous activity and process of change [71].

### 2.4. Methodological quality and risk of bias

The quality of the evidence from cross-sectional and longitudinal studies was evaluated using the Grading of Recommendations, Assessment, Development and Evaluations (GRADE) [72]. In accordance with the GRADE ProGDT online software, evidence was classified into high quality, moderate quality, low quality, and very low quality [73].

The risk of bias in quantitative studies was analyzed using the 27-item Downs and Black checklist [74]. As some items of this checklist were not applicable to observational study designs, a shorter version, adapted from a previous study, was used for cross-sectional (0–12 points) and longitudinal (0–16 points) designs [75]. Therefore, a subset of 16 questions (corresponding to Questions 1–3, 5–7, 9–12, 17, 18, 20, 21, 25, 26) was used. The score for each study was calculated as a percentage of the total score, and scores above 70% were considered “low risk of bias,” while scores below 70% were considered “high risk of bias” [74].

The quality of evidence and the risk of bias in qualitative studies was classified using the 10-item Critical Appraisal Skills Program (CASP) qualitative research checklist [76]. The overall scores were classified as low quality (one star; 0–3 points), medium quality (two stars; 4–7 points), and high quality (three stars; 8–10 points) [77].

For all studies, information on the declaration of potential conflict of interests and ethical approval was extracted. The analysis of the quality of the evidence and bias risk was performed independently by two trained reviewers (RMFS and CRM), and disagreements were resolved by a third reviewer (MN). The reviewers were trained in the use of instruments to analyze quality of evidence and bias risk before beginning their assessment [78].

## 3. Results

### 3.1. Description of the selected studies

A total of 6,384 records were imported after searching literature in five databases. Of these, 2,586 duplicates were removed, and 3,658 were excluded based on title and abstract screening, leaving 140 studies for full-text assessment. Eighty-one irrelevant studies were excluded, leaving 59 studies for inclusion in the review (37 on high school students and 22 on university students) (**Fig 1**). No studies were found through secondary (i.e., reference) searching.

**Fig 2:**
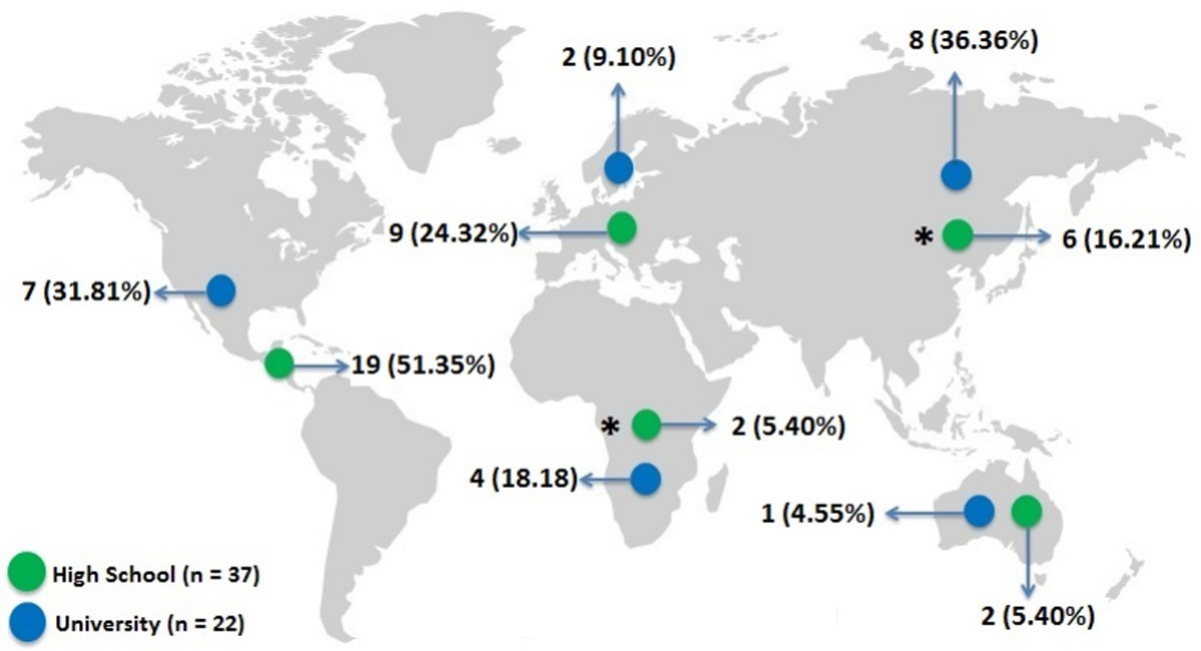
Total number of studies per geographic region (*one study on high school students was carried out in two continents). Figure available at https://br.freepik.com/vetores-gratis/.

The studies were published between 1989 and 2021, with a majority published after 2010 (25 [67.5%] on high school and 17 [77.2%] on university students). Overall, the included studies were conducted in 31 countries (high school student studies: 23 countries, and university student studies: 15 countries). Studies on high school students were predominantly conducted in North America and Europe, whereas studies on university students were predominantly from Asia and North America. The details of studies per geographic region are presented in **Fig 2**.

The sample size in the studies ranged between 20 and 5,663. Sixteen (43.2%) studies on high school students [73–88] and 10 (45.5%) on university students [89–98] had participants ranging from 100–500. The age range for high school students was 10–16 years in 24 (64.8%) studies [79, 81, 82, 85, 86, 99–117] and 17–25 years for university students in 19 (86.3%) studies [89, 90, 92, 93, 95–98, 118–128]. Twenty-eight (75.6%) studies on high school students [79, 80, 82, 83, 85, 86, 88, 99–107, 113–117, 129–134, 138] and 17 (77.2%) on university students [89–92, 94, 95, 97, 98, 107, 108, 118–120, 126–128] consisted of participants of both sexes. Seven (18.9%) studies on high school students [81, 108–112] and five (22.7%) on university students [93, 96, 121–123] included exclusively female participants.

The most commonly used study design was cross-sectional, used in 24 (64.8%) studies on high school [79–83, 87, 88, 99–102, 108–110, 116, 117, 129–135, 138] and 17 (77.2%) on university students [89–96, 118–121, 124, 125, 127, 128, 136]. The most frequently used methods for data collection were: questionnaires for 25 (67.5%) studies on high school and 17 (77.2%) for university students, followed by interviews, used in 10 (27.0%) studies on high school and five (22.7%) on university students. Questionnaires developed by the authors themselves were used in 11 (29.7%) studies on high school and six (27.2%) on university students.

The questionnaires examining barriers to physical activity in high school students were the Barriers to Physical Activity Questionnaire (n = 4, 10.8%) [129, 131, 134, 135] and Perceived Barriers to Physical Activity Questionnaire (n = 2, 5.4%) [79, 116]. The questionnaires examining barriers to physical activity in university students were the Exercise Benefits/Barriers Scale (n = 5, 22.7%) [89, 92, 94, 120, 121], A List of Possible Barriers to Physical Activity (n = 2, 9.0%) [127, 128] and Barriers to Being Active (n = 2, 9.0%) [118, 136, 137]. The detailed characteristics of the studies on high school and university students are shown in **Tables 1–3**.

**Table 1:** Characteristics of studies on high school students and university students.

Characteristics	Categories	High school students n (%)	University students n (%)
**Publication Year**	Prior to 2001	2 (5.41%)	0 (0.00%)
	2002−2010	10 (27.03%)	5 (22.73%)
	2011−2021	25 (67.57%)	17 (77.27%)
Region* Africa	Algeria	1 (2.70%)	0 (0.00%)
	Egypt	0 (0.00%)	2 (9.09%)
	Libya	1 (2.70%)	0 (0.00%)
	Morocco	1 (2.70%)	0 (0.00%)
	South Africa	0 (0.00%)	2 (9.09%)
America	United States of America	7 (18.92%)	4 (18.18%)
	Canada	5 (13.51%)	1 (4.55%)
	Brazil	6 (16.22%)	1 (4.55%)
	Colombia	0 (0.00%)	1 (4.55%)
	Uruguay	1 (2.70%)	0 (0.00%)
Asia	India	1 (2.70%)	2 (9.09%)
	Iran	1 (2.70%)	0 (0.00%)
	Jordan	1 (2.70%)	0 (0.00%)
	Kuwait	1 (2.70%)	0 (0.00%)
	Oman	1 (2.70%)	0 (0.00%)
	Palestine	1 (2.70%)	0 (0.00%)
	Malaysia	1 (2.70%)	0 (0.00%)
	Syria	1 (2.70%)	0 (0.00%)
	United Arab Emirates	1 (2.70%)	1 (4.55%)
	Turkey	1 (2.70%)	0 (0.00%)
	China	0 (0.00%)	1 (4.55%)
	Pakistan	0 (0.00%)	1 (4.55%)
	Saudi Arabia	0 (0.00%)	2 (9.09%)
	Thailand	0 (0.00%)	1 (4.55%)
Europe	United Kingdom	3 (8.11%)	0 (0.00%)
	Spain	4 (10.81%)	0 (0.00%)
	Poland	1 (2.70%)	1 (4.55%)
	Italy	1 (2.70%)	0 (0.00%)
	Denmark	0 (0.00%)	1 (4.55%)
Oceania	Australia	1 (2.70%)	1 (4.55%)
	New Zealand	1 (2.70%)	0 (0.00%)
**Sex**	Both sexes	28 (75.68%)	17 (77.27%)
	Female sex only	7 (18.92%)	5 (22.73%)
	Male sex only	2 (5.41%)	0 (0.00%)
**Main Result (barriers)**	Lack of time	16 (43.24%)	11 (50.00%)
	Lack of social support	14 (37.84%)	3 (13.63%)
	Lack of accessible	7 (18.92%)	3 (13.63%)
	Lack of motivation	6 (16.22%)	4 (18.18%)

*the total is higher than 100% because one study with university students was carried out in seven countries.

**Table 2:** Characteristics of the studies examining barriers to physical activity in high school students.

Author (year) Country	Participants N (% male)	Age (mean or range)	*Barriers Dimensions	Main Results (barriers)
**Cross-sectional (n = 24)**				
Allison et al. (1999), Canada [79]	1,041 (51%)	14.9 years (mean)	PEC; SC	Time constraints due to school work (p = 0.004); Other interests (p = 0.001); Family activities (p = 0.001).
Akpinar (2020), Turkey [80]	384 (51%)	13–19 years	PEC; SC; EN	Lack of time; Lack of support; Safety issues.
Camargo et al. (2021), Brazil [131]	1,518 (40%)	15–18 years	PEC; SC; EN	Laziness, not having company and climate.
Dambros et al. (2011), Brazil [87]	424 (54%)	14–18 years	PEC; SC; EN	Time devoted to studies, absence of an exercise partner, poor weather and long work hours.
Dias et al. (2015), Brazil [135]	1,049 (60%)	14–19 years	PEC; EN	Prefer to do other things (p = 0.003); Feel lazy (p = < 0.001); Lack of facilities nearby (p = 0.01); Lack of motivation (p = < 0.001); So much homework (p = 0.01).
Fahlman et al. (2006), USA [109]	1,314 (0%)	16.2 ± 0.9 years	PAC; EN	Physical activity makes sweat too much or makes tired, safety issues in neighborhood
Fernandez et al. (2017), Spain [88]	143 (53%)	14–17 years	PEC	Life demands and lack of time (p = 0.113); Tiredness and laziness (p = 0.001); Body image (p = 0.001).
Garcia et al. (2011), Brazil [138]	118 (43%)	10–19 years	SC; EN	Lack of company or friends; Lack of places were adequate.
Gunnell et al. (2015), Canada [117]	507 (44%)	12.1 ± 0.6 years	PEC; EN	External (not having equipment); Internal (lack of interest in physical activity).
Hsu et al. (2011), USA [116]	350 (21%)	12.5 ± 0.6 years	PEC; SC	External (lack of family social support and peer (friend) support, family responsibility); Internal (lack of self-discipline, willpower, illness, disability, injury).
Jodkowska et al. (2015), Poland [102]	3,346 (47%)	10–16 years	PEC; SC	Boys (p < 0.001): lack of time, skills, willpower and support; Girls (p < 0.001): lack of skills, energy, support and time.
Musaiger et al. (2013), Algeria, Jordan, Kuwait, Libya, Palestine, Syria and the United Arab Emirates, [132]	4,698 (47%)	15–18 years	PEC; SC	Lack of motivation to do physical activity; Less support from teachers; Lack of time to do physical activity.
Pandolfo et al. (2016), Brazil [129]	348 (53%)	14–19 years	PEC; EN	Lack of time (p = 0.001); Adverse weather conditions (p = 0.002).
Padehban et al. (2018), Iran [99]	280 (54%)	13–15 years	PEC; SC; EN	Lack of relatives supports (53.6%); To being far from sports places (35%); Lack of enough self-confidence (33.2%).
Portela-Pinto et al. (2019), Spain [133]	852(49%)	12–17 years	PEC	Fatigue or laziness.
Robbins et al.(2003), USA [110]	77 (0%)	11–14 years	PEC	Ashamed of physical appearance when exercising and lack of motivation.
Robbins et al. (2009), USA [100]	206 (50%)	11–14 years	PEC	Minor aches and pains from activity 2.29 ± 1.04; Tiredness 2.26 ± 1.01; Too busy 2.18 ± 1.07.
Rosselli et al. (2020), Italy [130]	368(58%)	18.3 ± 0.7 years	PEC	Lack of time; Lack of energy; Lack of willpower.
Santos et al.(2010), Brazil [134]	1,609 (40%)	14–18 years	PEC; SC	Lack of relatives supports; laziness and prefer to do other things.
Serrano et al. (2017), Spain [101]	248 (48%)	15.3 ± 1.8 years	PEC	Lack of time.
Sherar et al. (2009), United Kingdom [81]	221 (0%)	15.3 ± 0.63 years	PEC; EN	Lack of motivation\ lazy; Paid work; Illness or injury.
Tappe et al. (1989), USA [82]	236 (41%)	15.9 years (mean)	PEC; EN	Time constraints (p = 0.052); Unsuitable weather (p = 0.056); Interest or desire (p = 0.084); School and schoolwork (p = 0.092).
Youssef et al. (2013), Oman [83]	439 (48%)	15–18 years	PEC; SC; EN	Other recreational activities more entertaining (72.2%); Having limited energy to exercise (43.3%); Thinking that exercise was difficult and too tiring (40.1%); Agreed that parents give priority to academic success (71.5%); Not having leisure time due to academic responsibilities (65.4%).
Zaragoza et al. (2011), Spain [108]	714 (0%)	12–15 years	PEC; EN	Do not like physical activity (p = 0.001); Are not good at physical activity sports (p = 0.001); Lazy to do physical activity (p = 0.001); Insecurity doing outdoor physical activity (p < 0.001); There is no one to do physical activity (p < 0.001).
**Longitudinal (n = 1)**				
Eime et al. (2015), Australia [84]	440 (0%)	11–18 years	PEC	Lack of energy (p = 0.047); Lack of time due to other leisure activities (p = 0.006).
**Qualitative (n = 12)**				
Abdelghaffar et al. (2019), Morocco [113]	46 (50%)	14–16 years	PEC; SC; EN	Intrapersonal (e.g., motivating and limiting factors, physical activity awareness, and time constraints); Interpersonal/cultural (e.g., social support and gender and cultural norms); Environmental (e.g., access to opportunities).
Allison et al. (2005), Canada [103]	26 (100%)	15–16 years	PEC; SC; EN	External (e.g., influence of peers and family, issues of inaccessibility); Internal (e.g., television watching and computer and internet use).
Bélanger et al. (2011), Canada [85]	165 (35%)	10–12 years	EN	Lack of access.
Butt et al., (2011), United Kingdom [104]	1,163 (39%)	13–16 years	PEC; PAC	Lack of time and physical exertion.
Dwyer et al. (2006), Canada [111]	73 (0%)	15–16 years	PEC; SC; EN	Lack of time, involvement in technology-related activities, influence of peers, concern about safety and inaccessibility of facilities.
Hohepa et al. (2006), New Zealand [114]	44 (45%)	13–15 years	PEC; SC; EN	Lack of peer social support and low accessibility to, and availability of, physical activity opportunities.
Moore et al. (2010), USA [115]	50 (44%)	12.1 years (mean)	SC; EN	School policies, crime or danger.
Parobii et al. (2018), Uruguay [105]	65 (47%)	11–15 years	PEC; SC; EN	Lack of access and availability of physical activity opportunities both within and outside of school time, lack of places as well as equipment and infrastructure for engagement in physical activity, and lack of time and competing activities such as video games.
Robbins et al. (2010), USA [106]	40 (100%)	11–13 years	EN	Lack of equipment and places for physical activity.
Satija et al. (2018), India [86]	174 (47%)	12–16 years	PEC; SC; EN	Negative consequences of physical activity participation; Disapproval for participating in physical activity; Reduced opportunity for physical activity in schools.
Sharif Ishak et al. (2020), Malaysia [107]	72 (51%)	13–14 years	PEC; SC; EN	Time constraint, no motivation, physically unwell or tired, no companion, security issue at playground or exercise facilities, or venue, and weather.
Wetton et al. (2013), United Kingdom [112]	60 (0%)	15–16 years	PEC; SC	Internal factors (e.g., lack of ability and lack of enjoyment), Existing stereotypes (e.g., boys will always be better in sport, family context, media), Other hobbies (e.g., lack of time, prefers cooking and other artistic activities) and Teachers (e.g., lack of attention the teachers, always praise the best students).

*PEC: Psychological, Emotional and Cognitive; EN: Environmental; SC: Sociocultural; PAC: Physical Activity Characteristics.

**Table 3:** Characteristics of the studies examining barriers to physical activity in university students.

Author (year), Country	Participants N (% male)	Age (mean or range)	*Barrier Dimensions	Main Results (barriers)
**Cross-sectional (n = 17)**				
Awadalla et al. (2014), Saudi Arabia [127]	1,257 (34%)	17–25 years	PEC; EN	Lack of safe sports places (p = 0.004).
Chan (2014), China [89]	193 (35%)	20.1 ± 1.3 years	PEC; PAC	Fatigue brought on by exercising, lack of time.
El-Bagoury et al. (2017), Egypt [90]	445 (41%)	20.3 ± 1.5 years	PEC	Lack of time.
El-Gilany et al. (2011), Egypt [128]	1,708 (50%)	17–25 years	PEC; EN	Lack of time; Lack of accessible and suitable sports place; Lack of safe sports places.
Frederick et al. (2020), USA [120]	862 (22%)	20.1 ± 1.4 years	PEC; EN; PAC	Lack of accessible and suitable sports place; Lack of time; Lack of support; Physical exertion.
Gawwad (2008), Saudi Arabia [91]	302 (50%)	20–26 years	PEC; EN	Lack of time and resources.
Grubbs et al. (2002), USA [92]	147 (18%)	18–24 years	PEC; PAC	Lack of time (2.79 ± 0.66); Physical exertion (2.71 ± 0.67).
Gyurcsik et al. (2004), Canada [93]	132 (0%)	17–19 years	PEC; EN	School workload too high to allow for physical activity, job cuts into physical activity time, weather is too cold and gets dark too early.
Kgokong et al. (2020), South Africa [94]	296 (17%)	18–29 years	PAC	Physical exertion.
Kulavic et al. (2013), USA [118]	746 (40%)	19.1 ± 1.2 years	PEC; EN	Fear of injury (p = 0.001); Lack of resources (p = 0.017); Lack of skill (p = 0.003).
Nishimwe-Niyimbanira et al. (2014), South Africa [124]	540 (46%)	19.9 ± 2 years	PEC; PAC	Physical exertion (p < 0.001); Time expenditure (p = 0.007).
Ramirez-Velez (2015), Colombia [136]	5,663 (59%)	18–30 years	PEC; EN	Fear of injury (87.0%); Lack of skill (79.8%); Lack of resources (64.3%).
Samara et al. (2015), Denmark [121]	94 (0%)	18–22 years	EN	Lack of designated areas available for physical activity (75.0%).
Silliman et al. (2004), USA [95]	471 (40%)	18–25 years	PEC	Lack of time (36.30%); Lack of motivation (21.86%).
Sousa et al. (2013), Brazil [119]	1,083 (45%)	17–23 years	PEC; SC; EN	Uncomfortable climate, overwork, family and study obligations.
Sukys et al. (2019), Poland [125]	709 (56%)	18–25 years	PEC; SC	Lack of support (2.56 ± 1.11); Lack of motivation (2.15 ± 0.97).
Vaz et al. (2003), India [96]	259 (0%)	20 ± 3 years	PEC	Lack of time (p = 0.290); Lack of motivation (p = 0.570).
**Longitudinal (n = 1)**				
Ranasinghe et al. (2016), Australia [97]	113 (33%)	20–25 years	PEC	Lack of time, lack of motivation and lack willpower.
**Qualitative (n = 4)**				
Anjali et al. (2018), India [126]	67 (28%)	18–24 years	PEC; SC; EN	Lack of time, constraint, tiredness, stress, family control, safety issues.
Burton et al. (2021), United Arab Emirates [122]	25 (0%)	18–25 years	SC	Lack of support.
Laar et al. (2019), Pakistan [123]	20 (0%)	19–24 years	SC; EN	Limitations of socioeconomic factors, religious values, and culture.
Wattanapisit et al. (2016), Thailand [98]	279 (37%)	20.9 ± 1.8 years	PEC	Study-related activities and overtime shift work.

*PEC: Psychological, Emotional and Cognitive; EN: Environmental; SC: Sociocultural; PAC: Physical Activity Characteristics.

For both high school and university students, the most frequently perceived barriers to physical activity were in the 1) psychological, emotional, and cognitive; 2) environmental; and 3) sociocultural categories. In particular, the psychological, emotional, and cognitive barriers were the most frequently reported in both quantitative and qualitative studies. In studies on high school students, 32 (86.4%) barriers belonged to the psychological, emotional, and cognitive category, whereas for university students, 18 (81.8%) corresponded to this category. **Table 4** presents the main barriers (factors) for each category according to study design.

**Table 4:** Main barriers for each dimension grouped by the study design.

High school students		Undergraduate university students	
Dimensions	Barriers	Dimensions	Barriers
**Cross-sectional**			
(n = 24)		(n = 17)	
PEC	Lack of time [75, 76, 78, 84, 86, 95, 129, 134, 138]; Lack of willpower [95, 112, 138]; Lack of motivation [77, 106, 134, 135]	PEC	Lack of time [91, 92, 99, 101, 116, 120, 124, 130]; Lack of motivation [91, 92, 121]
EN	Lack of accessible [135]	EN	Lack of accessible [116, 117, 124]
SC	Lack of social support [76, 83, 87, 95, 112, 126, 131, 134]	SC	Lack of social support [116, 121]
**Longitudinal**			
(n = 1)		(n = 1)	
PEC	Lack of time [84];	PEC	Lack of time [97]; Lack of willpower [97]; Lack of motivation [97]
**Qualitative**			
(n = 12)		(n = 4)	
PEC	Lack of time [96–98, 103, 107, 108]; Lack of motivation [103, 109]	PEC	Lack of time [94, 122]
EN	Lack of accessible [81, 96, 98, 107, 109, 110]		
SC	Lack of social support [96, 103, 107–110]	SC	Lack of social support [122]

*PEC: Psychological, Emotional and Cognitive; EN: Environmental; SC: Sociocultural.

### 3.2 Quality of studies and risk of bias

Thirty-four (91.8%) studies on high school students and 19 (86.3%) on university students had explicitly stated that they sought ethical approval. Conflicts of interest were declared in 10 (27.0%) studies on high school students and 10 (45.4%) on university students. The quality of the evidence for 16 (66.6%) studies on high school students and 15 (88.2%) on university students, using the cross-sectional and/or longitudinal design, was classified as “low quality.” Sixteen qualitative studies had high methodological quality. Most studies on high school students had a low risk of bias (i.e., they had scores above 70%), whereas most studies on university students had a high risk of bias (i.e., they had scores below 70%). The description for the quality of studies and risk of bias is presented in **Tables 5 and 6.**

**Table 5:** Methodological quality and strength of evidence for studies examining barriers to physical activity in high school students.

			**Downs and Black checklist**																		**GRADE**
**Quantitative study (year)**	**Conflict of interests**	**Ethical approval**	**A**	**B**	**C**	**D**	**E**	**F**	**G**	**H**	**I**	**J**	**K**	**L**	**M**	**N**	**O**	**P**	**Total**	**Score**	
**Cross-sectional (n = 24)**																					
Allison et al. [79]	*	Yes	1	1	1	0	1	1	-	1	1	1	-	1	1	-	0	-	10/12	83%	●●●○
Akpinar [80]	No	Yes	1	1	1	0	1	1	-	1	1	0	-	1	1	-	0	-	09/12	75%	●●○○
Camargo et al. [131]	*o	Yes	1	1	1	0	1	1	-	1	1	0	-	1	1	-	0	-	09/12	75%	●●●○
Dambros [87]	*	Yes	1	1	1	0	1	1	-	1	1	1	-	1	1	-	0	-	10/12	83%	●●○○
Dias et al. [135]	*	Yes	1	1	1	0	1	1	-	1	1	1	-	1	1	-	0	-	10/12	83%	●●●○
Fahlman et al. [109]	*	Yes	1	1	1	0	1	1	-	1	1	1	-	1	1	-	0	-	10/12	83%	●●●○
Fernandez et al. [88]	*	Yes	1	1	1	0	1	1	-	1	1	1	-	1	1	-	0	-	10/12	83%	●●○○
Garcia et al. [138]	*	Yes	1	1	1	0	1	1	-	1	1	1	-	1	1	-	0	-	10/12	83%	●○○○
Gunnell et al. [117]	No	Yes	1	1	1	0	1	1	-	1	1	1	-	1	1	-	0	-	10/12	83%	●●○○
Hsu et al. [116]	*	Yes	1	1	1	0	1	1	-	1	1	1	-	1	1	-	0	-	10/12	83%	●●○○
Jodkowska et al. [102]	*	Yes	1	1	1	0	1	1	-	1	1	1	-	1	1	-	0	-	10/12	83%	●●●○
Musaiger et al. [132]	*	Yes	1	1	1	0	1	1	-	1	1	1	-	1	1	-	0	-	10/12	83%	●●●○
Pandolfo et al. [129]	*	Yes	1	1	1	0	1	1	-	1	1	0	-	1	1	-	0	-	09/12	75%	●●○○
Padehban et al. [99]	No	Yes	1	1	1	0	1	1	-	1	1	1	-	1	1	-	0	-	10/12	83%	●○○○
Portela-Pinto et al. [133]	No	Yes	1	1	1	0	1	1	-	1	1	0	-	1	1	-	0	-	09/12	75%	●●○○
Robbins et al. [110]	*	Yes	1	1	1	0	1	1	-	1	1	0	-	1	1	-	0	-	09/12	75%	●●○○
Robbins et al. [100]	*	Yes	1	1	1	0	1	1	-	1	1	0	-	1	1	-	0	-	09/12	75%	●●○○
Rosselli et al. [130]	No	Yes	1	1	1	0	1	1	-	1	1	0	-	1	1	-	0	-	09/12	75%	●●○○
Santos et al. [134]	*	Yes	1	1	1	0	1	1	-	1	1	0	-	1	1	-	0	-	09/12	75%	●●●○
Serrano et al. [101]	*	*	1	1	1	0	1	1	-	1	1	0	-	1	1	-	0	-	09/12	75%	●●○○
Sherar et al. [81]	*	Yes	1	1	1	0	1	1	-	1	1	0	-	1	1	-	0	-	09/12	75%	●●○○
Tappe et al. [82]	*	*	1	1	1	0	1	1	-	1	1	0	-	1	1	-	0	-	09/12	75%	●●○○
Youssef et al. [83]	*	Yes	1	1	1	0	1	1	-	1	1	0	-	1	1	-	0	-	09/12	75%	●●○○
Zaragoza et al. [108]	*	Yes	1	1	1	0	1	1	-	1	1	1	-	1	1	-	0	-	10/12	83%	●●○○
**Longitudinal (n = 1)**																					
Eime et al. [84]	*	Yes	1	1	1	0	1	1	0	1	1	1	1	0	1	1	0	0	11/16	68%		●●○○
**Qualitative study (n = 12)**	**Conflict of interests**	**Ethical approval**																		**CASP**	
Abdelghaffar et al. [113]	*	Yes	NA																		☆☆☆
Allison et al. [103]	No	Yes	NA																		☆☆☆
Bélanger et al. [85]	*	Yes	NA																		☆☆☆
Butt et al. [104]	*	Yes	NA																		☆☆☆
Dwyer et al. [111]	*	Yes	NA																		☆☆☆
Hohepa et al. [114]	No	*	NA																		☆☆☆
Moore et al. [115]	*	Yes	NA																		☆☆
Parobii et al. [105]	*	Yes	NA																		☆☆☆
Robbins et al. [106]	No	Yes	NA																		☆☆☆
Satija et al. [86]	*	Yes	NA																		☆☆☆
Sharif Ishak et al. [107]	*	Yes	NA																		☆☆☆
Wetton et al. [112]	No	Yes	NA																		☆☆☆

**Downs and Black checklist**: A) objective clearly stated; B) main outcomes clearly described; C) sample characteristics clearly defined; E) main findings clearly defined; F) random variability in estimates provided; G) lost to follow-up described; H) probability values reported; I) sample target representative of population; J) sample recruitment representative of population; L) study based on “data dredging,” if applied; N) statistical tests used appropriately; and O) primary outcomes valid/reliable; (correspond to questions 1–3, 6–7, 9–12, 16, 18, 20).

* not reported. NA, not applicable.

**GRADE**: Grading of Recommendations, Assessment, Development and Evaluations, where cross-sectional and longitudinal studies with one filled circle = very low quality, two filled circles = low quality, three filled circles = moderate quality, and four filled circles = high quality.

**CASP**: Critical Appraisal Skills Programme Qualitative Research Checklist, where qualitative studies were classified as low (one star: 0–3 points), medium (two stars: 4–7 points), and high quality (three stars: 8–10 points).

**Table 6:** Methodological quality and strength of evidence for studies examining barriers to physical activity in undergraduate university students.

			**Downs and Black checklist**																		**GRADE**
**Study (year)**	**Conflict of interests**	**Ethical approval**	**A**	**B**	**C**	**D**	**E**	**F**	**G**	**H**	**I**	**J**	**K**	**L**	**M**	**N**	**O**	**P**	**Total**	**Score**	
**Cross-sectional (n = 17)**																					
Awadalla et al. [127]	No	Yes	1	1	1	0	1	1	-	1	1	1	-	1	1	-	0	-	10/12	83%	●○○○
Chan [89]	*	Yes	1	1	1	0	1	0	-	1	1	0	-	1	1	-	0	-	08/12	66%	●●○○
El-Bagoury et al. [90]	No	Yes	1	1	1	0	1	0	-	1	1	0	-	1	1	-	0	-	08/12	66%	●●○○
El-Gilany et al. [128]	*	Yes	1	1	1	0	1	0	-	1	1	0	-	1	1	-	0	-	08/12	66%	●●○○
Frederick et al. [120]	*o	Yes	1	1	1	0	1	0	-	1	1	1	-	1	1	-	0	-	09/12	75%	●●○○
Gawwad [91]	*	Yes	1	1	1	0	1	1	-	1	1	1	-	1	1	-	0	-	10/12	83%	●●○○
Grubbs et al. [92]	*	Yes	1	1	1	0	1	1	-	1	1	0	-	1	1	-	0	-	09/12	75%	●●○○
Gyurcsik et al. [93]	*	Yes	1	1	1	0	1	0	-	1	1	0	-	1	1	-	0	-	08/12	66%	●●○○
Kgokong et al. [94]	*	*	1	1	1	0	1	0	-	1	1	0	-	1	1	-	0	-	08/12	66%	●●○○
Kulavic et al. [118]	No	Yes	1	1	1	0	1	0	-	1	1	1	-	1	1	-	0	-	09/12	75%	●●○○
Nishimwe-Niyimbanira et al. [124]	*	Yes	1	1	1	0	1	0	-	1	1	0	-	1	1	-	0	-	08/12	66%	●●○○
Ramirez-Velez [136]	*	*	1	1	1	0	1	0	-	1	1	1	-	1	1	-	0	-	09/12	75%	●●○○
Samara et al. [121]	*	Yes	1	1	1	0	1	0	-	1	1	0	-	1	1	-	0	-	08/12	66%	●●○○
Silliman et al. [95]	No	Yes	1	1	1	0	1	0	-	1	1	0	-	1	1	-	0	-	08/12	66%	●○○○
Sousa et al. [119]	*	Yes	1	1	1	0	1	0	-	1	1	1	-	1	1	-	0	-	09/12	75%	●●○○
Sukys et al. [125]	No	Yes	1	1	1	0	1	0	-	1	1	0	-	1	1	-	0	-	08/12	66%	●●○○
Vaz et al. [96]	*	*	1	1	1	0	1	0	-	1	1	0	-	1	1	-	0	-	08/12	66%	●●○○
**Longitudinal (n = 1)**																					
Ranasinghe et al. [97]	No	Yes	1	1	1	0	1	0	-	1	1	0	-	1	1	-	0	-	08/12	66%	●●○○
**Qualitative study (n = 4)**	**Conflict of interests**	**Ethical approval**																			**CASP**
Anjali et al. [126]	*	Yes		NA																	☆☆☆
Burton et al. [122]	No	Yes		NA																	☆☆☆
Laar et al. [123]	No	Yes			NA																☆☆☆
Wattanapisit et al. [98]	No	Yes			NA																☆☆☆

**Downs and Black checklist**: A) objective clearly stated; B) main outcomes clearly described; C) sample characteristics clearly defined; E) main findings clearly defined; F) random variability in estimates provided; G) lost to follow-up described; H) probability values reported; I) sample target representative of population; J) sample recruitment representative of population; L) study based on “data dredging,” if applied; N) statistical tests used appropriately; and O) primary outcomes valid/reliable; (correspond to questions 1–3, 6–7, 9–12, 16, 18, 20).

* not reported. NA, not applicable.

**GRADE**: Grading of Recommendations, Assessment, Development and Evaluations, where cross-sectional and longitudinal studies with one filled circle = very low quality, two filled circles = low quality, three filled circles = moderate quality, and four filled circles = high quality.

**CASP**: Critical Appraisal Skills Programme Qualitative Research Checklist, where qualitative studies were classified as low (one star: 0–3 points), medium (two stars: 4–7 points) and high quality (three stars: 8–10 points).

## 4. Discussion

This systematic review summarizes the findings of qualitative and quantitative research on barriers to physical activity and their dimensions in high school and university students. A total of 38,319 adolescents and young adults from 31 countries were part of the studies included in our review. The main barriers identified in high school and university students were lack of time, lack of motivation, and lack of accessible places.

The findings of the current review suggest that psychological, emotional, and cognitive factors were the most examined in quantitative studies (92.0% of studies with high school students and 94.0% with university students), whereas environmental (83.3% of studies with high school students) and sociocultural (75.0% of studies with university students) factors were most frequently studied in qualitative studies. Furthermore, the main barriers to physical activity in high school students were related to the following dimensions: psychological, emotional, and cognitive (lack of time and motivation); sociocultural (lack of social support); and environmental (lack of accessible places). Previous studies have also identified these barriers and dimensions as the most common [139–141]. In addition, a recent systematic review identified these dimensions as the most common in terms of barriers to physical activity in adolescents [57]. For the environmental dimension, a previous study suggested that schools must work with community partners and officials to provide environments that optimally support physical activity in adolescent students [142].

The main barriers to physical activity in undergraduate university students were related to the following dimensions: psychological, emotional, and cognitive (lack of time and motivation); environmental (lack of accessible places); and socioeconomic and demographic (lack of financial resources). Barriers in the psychological, emotional, and cognitive category were identified in almost all parts of the world that were covered by the included studies. Among others, lack of time was the most cited barrier to physical activity in university students. Although no previous systematic reviews have identified barriers to physical activity among university students, some qualitative studies have shown the presence of motivational and time-related barriers as factors preventing university students from practicing physical activity [122, 143, 144]. Furthermore, barriers to physical activity are almost similar in reviews on different populations, for example in individuals from the Middle East and North Africa [145], pregnant women [146] and medical services professionals [147]. A recent systematic review showed that cultural values (e.g., general and gender norms) affect the practice of physical activity in specific countries (e.g., Arab countries) [148]. Further, it is important to note that access to university is restricted by socioeconomic status: adolescents and young adults with a lower socioeconomic level have less access to higher education, which may also be related to a greater social and cultural barrier to physical activity. Furthermore, socioeconomic barriers permeate all other barriers. For example, motivation for physical activity, knowledge of its benefits, time availability, social support from family, and access to equipment are negatively influenced by socioeconomic vulnerability [149].

Many behavior change theories [150–155], health behavior adoption theories [156, 157], and social ecological models [158, 159] have been used to promote active lifestyles in different population groups. However, behavior change is a complex and multifaceted phenomenon with multiple levels of influence [152]. Therefore, multilevel physical activity interventions targeting several components (e.g., individuals, social and physical environments, and policies) have been shown to have promising effects [160–163]. Intrinsic motivation is an important factor used to determine active participation in physical activity and sport [35]; thus, to increase adolescents’ daily physical activity, special focus should be paid on increasing their intrinsic motivation [168]. Some studies have also pointed out the importance of context in understanding physical activity motivation and the role of culture in preventing participation in physical activity [160, 164–170].

Screen time was not identified as a barrier to physical activity, but it may be related to the “lack of time” barrier since spending more time on a device means having less time for other activities, including physical activity. A study with Spanish teenagers found that those who spent more time in front of screens spent less time performing physical activity [171]. In addition, screen time was reported as the main driver for adolescents’ inability to meet the recommendation of moderate-to-vigorous physical activity in the United Kingdom [172]. Understanding the barriers to physical activity is important because it may provide information useful for creating public health and educational policies. Thus, actions and programs to promote the practice of physical activity should always consider all dimensions of physical activity barriers, and special attention should be given to psychological, emotional, and cognitive factors.

The current study, as far as we know, is the first systematic review that summarizes the evidence (qualitative and quantitative) for barriers to physical activity practice in high school and university students. However, some limitations should be acknowledged. First, the heterogeneity across included studies did not allow a meta-analysis to be performed. Second, the majority of evidence on barriers to physical activity in high school and university students came from cross-sectional studies (69.49%), with two longitudinal studies. Third, there was a lack of standardization of instruments for identifying barriers to physical activity in students. Finally, gray literature was not included in the review. Therefore, future studies should be conducted with strong methodological rigor to generate better evidence, for example by using longitudinal designs, control bias, and a context-sensitive basis. The use of standardized global instruments for physical activity and barriers, mainly for university students, has also been advocated in a recent review [40].

## 5. Conclusion

The barriers to physical activity among high school and university students are mainly related to psychological, emotional, cognitive, environmental, and sociocultural factors. These findings suggest that future behavioral change interventions or interventions targeting barriers to physical activity should prioritize these dimensions. In addition, studies on the least explored dimensions (i.e., physical activity characteristics and behavioral attributes) are needed in the future.

## Supporting information

S1 Checklist Checklist PRISMA. (DOCX)

S1 Table Search strategy. (DOCX)

## References

[1] Martinez R, Lloyd-Sherlock P, Soliz P, Ebrahim S, Vega E, Ordunez P, et al. Trends in premature avertable mortality from non-communicable diseases for 195 countries and territories, 1990–2017: a population-based study. Lancet Glob Heal. 2020;8: e511–e523. Available from doi: 10.1016/S2214-109X(20)30035-8 32199120

[2] NCD Countdown 2030: pathways to achieving Sustainable Development Goal target 3.4. Lancet. 2020;396: 918–934. Available from: doi: 10.1016/S0140-6736(20)31761-X 32891217 PMC7470795

[3] Malta DC, Duncan BB, Schmidt MI, Teixeira R, Ribeiro ALP, Felisbino-Mendes MS, et al. Trends in mortality due to non-communicable diseases in the Brazilian adult population: National and subnational estimates and projections for 2030. Popul Health Metr. 2020;18: 1–14. Available from: doi: 10.1186/s12963-019-0201-0 32993685 PMC7525955

[4] Bell MF, Bayliss DM, Glauert R, Harrison A, Ohan JL. Chronic illness and developmental vulnerability at school entry. Pediatrics. 2016;137. Available from: doi: 10.1542/peds.2015-2475 27244787

[5] Anderson E, Durstine JL. Physical activity, exercise, and chronic diseases: A brief review. Sport Med Heal Sci. 2019;1: 3–10. Available from: 10.1016/j.smhs.2019.08.006 PMC9219321 35782456

[6] Ding D, Ramirez Varela A, Bauman AE, Ekelund U, Lee I-M, Heath G, et al. Towards better evidence-informed global action: lessons learnt from the Lancet series and recent developments in physical activity and public health. Br J Sports Med. 2020;54: 462 LP– 468. Available from: doi: 10.1136/bjsports-2019-101001 31562122 PMC7146932

[7] WHO. Global Action Plan on Physical Activity 2018–2030. 2018. Available from: https://www.cref6.org.br/wp-content/uploads/2018/09/Plano-Global.pdf

[8] Moeini B, Rezapur-Shahkolai F, Bashirian S, Doosti-Irani A, Afshari M, Geravandi A. Effect of interventions based on regular physical activity on weight management in adolescents: a systematic review and a meta-analysis. Syst Rev. 2021;10: 52. Available from: doi: 10.1186/s13643-021-01602-y 33557946 PMC7871535

[9] Chaput J-P, Klingenberg L, Rosenkilde M, Gilbert J-A, Tremblay A, Sjödin A. Physical Activity Plays an Important Role in Body Weight Regulation. Journal of Obesity. 2011. Available from: doi: 10.1155/2011/360257 20847894 PMC2931400

[10] Alidadi A, Jalili A. Relationship between physical fitness, body composition and blood pressure in active and passive students. Int J Pharm Biol Sci Arch. 2019. Available from: https://www.ijpba.in/index.php/ijpba/article/view/142

[11] Lombardi G, Ziemann E, Banfi G. Physical Activity and Bone Health: What Is the Role of Immune System? A Narrative Review of the Third Way. Front Endocrinol (Lausanne). 2019;10: 60. Available from: doi: 10.3389/fendo.2019.00060 30792697 PMC6374307

[12] Cruz-Jentoft AJ, Sayer AA. Sarcopenia. Lancet. 2019;393: 2636–2646. Available from: https://pubmed.ncbi.nlm.nih.gov/31171417/ doi: 10.1016/S0140-6736(19)31138-9 31171417

[13] Livingston G, Sommerlad A, Orgeta V, Costafreda SG, Huntley J, Ames D, et al. Dementia prevention, intervention, and care. Lancet. 2017;390: 2673–2734. Available from: https://pubmed.ncbi.nlm.nih.gov/28735855/ doi: 10.1016/S0140-6736(17)31363-6 28735855

[14] Tari AR, Norevik CS, Scrimgeour NR, Kobro-Flatmoen A, Storm-Mathisen J, Bergersen LH, et al. Are the neuroprotective effects of exercise training systemically mediated? Prog Cardiovasc Dis. 2019;62: 94–101. Available from: doi: 10.1016/j.pcad.2019.02.003 30802460

[15] Dale LP, Vanderloo L, Moore S, Faulkner G. Physical activity and depression, anxiety, and self-esteem in children and youth: An umbrella systematic review. Ment Health Phys Act. 2019;16: 66–79. Available from: 10.1016/j.mhpa.2018.12.001

[16] Donnelly JE, Hillman CH, Castelli D, Etnier JL, Lee S, Tomporowski P, et al. Physical Activity, Fitness, Cognitive Function, and Academic Achievement in Children: A Systematic Review. Med Sci Sport Exerc. 2016;48. Available from: https://www.ncbi.nlm.nih.gov/pmc/articles/PMC4874515/10.1249/MSS.0000000000000901 PMC4874515 27182986

[17] DeJonge ML, Omran J, Faulkner GE, Sabiston CM. University students’ and clinicians’ beliefs and attitudes towards physical activity for mental health. Ment Health Phys Act. 2020;18. Available from: 10.1016/j.mhpa.2019.100316

[18] Bherer L, Pothier K. Physical Activity and Exercise BT—Cognitive Training: An Overview of Features and Applications. In: Strobach T, Karbach J, editors. Cham: Springer International Publishing; 2021. pp. 319–330. Available from: 10.1007/978-3-030-39292-5_22

[19] Bull FC, Al-SS, Biddle S, Borodulin K, Buman MP, Cardon G, et al. World Health Organization 2020 guidelines on physical activity and sedentary behaviour. Br J Sports Med. 2020; 1451–1462. Available from: doi: 10.1136/bjsports-2020-102955 33239350 PMC7719906

[20] Memon AR, Stanton R, To Q, Schoeppe S, Urooj A, Alley S, et al. Sedentary behaviour research in adults: A scoping review of systematic reviews and meta-analyses. J Sports Sci. 2021;39: 2219–2231. Available from: doi: 10.1080/02640414.2021.1928382 34006177

[21] Tremblay MS, Aubert S, Barnes JD, Saunders TJ, Carson V, Latimer-Cheung AE, et al. Sedentary Behavior Research Network (SBRN)—Terminology Consensus Project process and outcome. Int J Behav Nutr Phys Act. 2017;14: 75. Available from: doi: 10.1186/s12966-017-0525-8 28599680 PMC5466781

[22] Memon AR, To QG, Vandelanotte C. Vigorously Cited: A Bibliometric Analysis of the 500 Most Cited Physical Activity Articles. J Phys Act Heal. 2021; 1–16. Available from: doi: 10.1123/jpah.2020-0744 34140424

[23] Vainshelboim B, Brennan GM, LoRusso S, Fitzgerald P, Wisniewski KS. Sedentary behavior and physiological health determinants in male and female college students. Physiol Behav. 2019;204: 277–282. Available from: doi: 10.1016/j.physbeh.2019.02.041 30831185

[24] Guthold R, Stevens GA, Riley LM, Bull FC. Worldwide trends in insufficient physical activity from 2001 to 2016: a pooled analysis of 358 population-based surveys with 1·9 million participants. Lancet Glob Heal. 2018;6: e1077–e1086. Available from: doi: 10.1016/S2214-109X(18)30357-7 30193830

[25] WHO. Orientation Programme on Adolescent Health for Health-care Providers—Handout New Modules. 2018. Available from: https://apps.who.int/iris/handle/10665/42868

[26] Guthold R, Stevens GA, Riley LM, Bull FC. Global trends in insufficient physical activity among adolescents: a pooled analysis of 298 population-based surveys with 1 · 6 million participants. Lancet Child Adolesc Heal. 2019;4: 23–35. Available from: doi: 10.1016/S2352-4642(19)30323-2 31761562 PMC6919336

[27] Hallal PC, Andersen LB, Bull FC, Guthold R, Haskell W, Ekelund U. Global physical activity levels: surveillance progress, pitfalls, and prospects. Lancet. 2012;380: 247–257. Available from: doi: 10.1016/S0140-6736(12)60646-1 22818937

[28] Corder K, Winpenny E, Love R, Brown HE, White M, Sluijs E van. Change in physical activity from adolescence to early adulthood: a systematic review and meta-analysis of longitudinal cohort studies. Br J Sports Med. 2019;53: 496–503. Available from: doi: 10.1136/bjsports-2016-097330 28739834 PMC6250429

[29] Lu C, Stolk RP, Sauer PJJ, Sijtsma A, Wiersma R, Huang G, et al. Factors of physical activity among Chinese children and adolescents: A systematic review. Int J Behav Nutr Phys Act. 2017;14: 1–10. Available from: doi: 10.1186/s12966-016-0456-9 28320408 PMC5360041

[30] Morseth B, Jørgensen L, Emaus N, Jacobsen BK, Wilsgaard T. Tracking of leisure time physical activity during 28 yr in adults: the Tromsø study. Med Sci Sports Exerc. 2011;43: 1229–1234. Available from: doi: 10.1249/MSS.0b013e3182084562 21131860

[31] Winpenny EM, Smith M, Penney T, Foubister C, Guagliano JM, Love R, et al. Changes in physical activity, diet, and body weight across the education and employment transitions of early adulthood: A systematic review and meta-analysis. Obes Rev. 2019;21: e12962. Available from: 10.1111/obr.12962 PMC7079102 31955496

[32] Gropper H, John JM, Sudeck G, Thiel A. The impact of life events and transitions on physical activity: A scoping review. PLoS One. 2020;15: e0234794–e0234794. Available from: doi: 10.1371/journal.pone.0234794 32569282 PMC7307727

[33] Dumith SC, Gigante DP, Domingues MR, Kohl HW 3rd. Physical activity change during adolescence: a systematic review and a pooled analysis. Int J Epidemiol. 2011;40: 685–698. Available from: doi: 10.1093/ije/dyq272 21245072

[34] Brooke HL, Corder K, Griffin SJ, van Sluijs EMF. Physical Activity Maintenance in the Transition to Adolescence: A Longitudinal Study of the Roles of Sport and Lifestyle Activities in British Youth. PLoS One. 2014;9: e89028. Available from: doi: 10.1371/journal.pone.0089028 24533167 PMC3923069

[35] Sierra-Díaz MJ, González-Víllora S, Pastor-Vicedo JC, López-Sánchez GF. Can We Motivate Students to Practice Physical Activities and Sports Through Models-Based Practice? A Systematic Review and Meta-Analysis of Psychosocial Factors Related to Physical Education. Frontiers in Psychology. 2019. p. 2115. Available from: https://www.frontiersin.org/article/10.3389/fpsyg.2019.02115 31649571 10.3389/fpsyg.2019.02115 PMC6795761

[36] Beauchamp MR, Puterman E, Lubans DR. Physical Inactivity and Mental Health in Late Adolescence. JAMA Psychiatry. 2018; 1–2. Available from: doi: 10.1001/jamapsychiatry.2018.0385 29710114

[37] Robazzi MLDCC. Promotion of physical and mental health and well-being in the university environment. Rev Eletrônica Saúde Ment Álcool e Drog. 2019;15: 1–3. Available from: 10.11606/issn.1806-6976.smad.2019.154951

[38] Fedewa M V, Das BM, Evans EM, Dishman RK. Change in weight and adiposity in college students: a systematic review and meta-analysis. Am J Prev Med. 2014;47: 641–652. Available from: doi: 10.1016/j.amepre.2014.07.035 25241201

[39] Vadeboncoeur C, Townsend N, Foster C. A meta-analysis of weight gain in first year university students: is freshman 15 a myth? BMC Obes. 2015;2: 22. Available from: doi: 10.1186/s40608-015-0051-7 26217537 PMC4511069

[40] Memon AR, Gupta CC, Crowther ME, Ferguson SA, Tuckwell GA, Vicent GE. Sleep and physical activity in university students: A systematic review and meta-analysis. Sleep Med Rev. 2021;58: 101482. Available from: doi: 10.1016/j.smrv.2021.101482 33864990

[41] Moraes ACF, Guerra PH, Menezes PR. The worldwide prevalence of insufficient physical activity in adolescents; a systematic review. Nutr Hosp. 2013;28: 575–584. Available from: doi: 10.3305/nh.2013.28.3.6398 23848074

[42] Hollis JL, Sutherland R, Williams AJ, Campbell E, Nathan N, Wolfenden L, et al. A systematic review and meta-analysis of moderate-to-vigorous physical activity levels in secondary school physical education lessons. Int J Behav Nutr Phys Act. 2017;14: 52. Available from: doi: 10.1186/s12966-017-0504-0 28438171 PMC5402678

[43] Irwin JD. Prevalence of university students’ sufficient physical activity: a systematic review. Percept Mot Skills. 2004;98: 927–943. Available from: doi: 10.2466/pms.98.3.927-943 15209309

[44] Keating XD, Guan J, Piñero JC, Bridges DM. A meta-analysis of college students’ physical activity behaviors. J Am Coll Health. 2005;54: 116–125. Available from: doi: 10.3200/JACH.54.2.116-126 16255324

[45] Pengpid S, Peltzer K, Kassean HK, Tsala Tsala JP, Sychareun V, Müller-Riemenschneider F. Physical inactivity and associated factors among university students in 23 low-, middle- and high-income countries. Int J Public Health. 2015;60: 539–549. Available from: doi: 10.1007/s00038-015-0680-0 25926342

[46] Cohen-Mansfield J, Marx MS, Guralnik JM. Motivators and Barriers to Exercise in an Older Community-Dwelling Population. J Aging Phys Act. 2003;11: 242–253. Available from: 10.1123/japa.11.2.242

[47] Bauman AE, Reis RS, Sallis JF, Wells JC, Loos RJF, Martin BW. Correlates of physical activity: why are some people physically active and others not? Lancet. 2012;380: 258–271. Available from: doi: 10.1016/S0140-6736(12)60735-1 22818938

[48] Reichert FF, Barros AJD, Domingues MR, Hallal PC. The role of perceived personal barriers to engagement in leisure-time physical activity. Am J Public Health. 2007;97: 515–519. Available from: doi: 10.2105/AJPH.2005.070144 17267731 PMC1805028

[49] Sallis JF, Cerin E, Conway TL, Adams MA, Frank LD, Pratt M, et al. Physical activity in relation to urban environments in 14 cities worldwide: a cross-sectional study. Lancet. 2016; 387(10034) 2207–2217. Available from: doi: 10.1016/S0140-6736(15)01284-2 27045735 PMC10833440

[50] Ferreira I, Van Der Horst K, Wendel-Vos W, Kremers S, Van Lenthe FJ, Brug J. Environmental correlates of physical activity in youth—A review and update. Obes Rev. 2007;8: 129–154. Available from: doi: 10.1111/j.1467-789X.2006.00264.x 17300279

[51] Seabra AF, Mendonça DM, Thomis MA, Anjos LA, Maia JA. Biological and socio-cultural determinants of physical activity in adolescents. Cad Saude Publica. 2008;24: 721–736. Available from: doi: 10.1590/s0102-311x2008000400002 18392349

[52] Van Der Horst K, Paw MJCA, Twisk JWR, Van Mechelen W. A brief review on correlates of physical activity and sedentariness in youth. Med Sci Sports Exerc. 2007;39: 1241–1250. Available from: doi: 10.1249/mss.0b013e318059bf35 17762356

[53] Sallis JF, Prochaska JJ, Taylor WC. A review of correlates of physical activity of children and adolescents. Med Sci Sports Exerc. 2000;32: 963–975. Available from: doi: 10.1097/00005768-200005000-00014 10795788

[54] Portela-Pino I, López-Castedo A, Martínez-Patiño MJ, Valverde-Esteve T, Domínguez-Alonso J. Gender differences in motivation and barriers for the practice of physical exercise in adolescence. Int J Environ Res Public Health. 2020;17. Available from: 10.3390/ijerph17010168 PMC6981955 31881707

[55] Vasquez T, Fernandez A, Haya-Fisher J, Kim S, Beck AL. A Qualitative Exploration of Barriers and Facilitators to Physical Activity Among Low-Income Latino Adolescents. Hisp Heal care Int Off J Natl Assoc Hisp Nurses. 2021;19: 86–94. Available from: doi: 10.1177/1540415320956933 32911975 PMC8496995

[56] Martins J, Marques A, Sarmento H, Carreiro Da Costa F. Adolescents’ perspectives on the barriers and facilitators of physical activity: A systematic review of qualitative studies. Health Educ Res. 2014;30: 742–755. Available from: 10.1093/her/cyv042 26324394

[57] Martins J, Costa J, Sarmento H, Marques A, Farias C, Onofre M, et al. Adolescents’ perspectives on the barriers and facilitators of physical activity: An updated systematic review of qualitative studies. Int J Environ Res Public Health. 2021;18. Available from: doi: 10.3390/ijerph18094954 34066596 PMC8125166

[58] Ferreira Silva RM, Mendonça CR, Noll M. Barriers to high school and university students’ physical activity: A systematic review protocol. Int J Educ Res. 2021;106: 2–6. Available from: 10.1016/j.ijer.2021.101743 PMC8979430 35377905

[59] Loprinzi PD, Cardinal BJ, Loprinzi KL, Lee H. Benefits and environmental determinants of physical activity in children and adolescents. Obes Facts. 2012;5: 597–610. Available from: doi: 10.1159/000342684 22986648

[60] Van Luchene P, Delens C. The Influence of Social Support Specific to Physical Activity on Physical Activity Among College and University Students: A Systematic Review. J Phys Act Heal. 2021;18: 737–747. Available from: doi: 10.1123/jpah.2020-0713 33883289

[61] Rech CR, de Camargo EM, de Araujo PAB, Loch MR, Reis RS. Perceived barriers to leisure-time physical activity in the Brazilian population. Rev Bras Med do Esporte. 2018;24: 303–309. Available from: 10.1590/1517-869220182404175052

[62] Page MJ, McKenzie JE, Bossuyt PM, Boutron I, Hoffmann TC, Mulrow CD, et al. The PRISMA 2020 statement: an updated guideline for reporting systematic reviews. BMJ. 2021;372: n71. Available from: doi: 10.1136/bmj.n71 33782057 PMC8005924

[63] Eriksen MB, Frandsen TF. The impact of patient, intervention, comparison, outcome (PICO) as a search strategy tool on literature search quality: a systematic review. J Med Libr Assoc. 2018;106: 420–431. Available from: doi: 10.5195/jmla.2018.345 30271283 PMC6148624

[64] Munn Z, Stern C, Aromataris E, Lockwood C, Jordan Z. What kind of systematic review should I conduct? A proposed typology and guidance for systematic reviewers in the medical and health sciences. BMC Med Res Methodol. 2018;18: 5. Available from: doi: 10.1186/s12874-017-0468-4 29316881 PMC5761190

[65] Lastella M, Halson SL, Vitale JA, Memon AR, Vincent GE. To Nap or Not to Nap? A Systematic Review Evaluating Napping Behavior in Athletes and the Impact on Various Measures of Athletic Performance. Nat Sci Sleep. 2021;13: 841–862. Available from: doi: 10.2147/NSS.S315556 34194254 PMC8238550

[66] Islam MS, Sujan MSH, Tasnim R, Sikder MT, Potenza MN, van Os J. Psychological responses during the COVID-19 outbreak among university students in Bangladesh. PLoS One. 2021;15: e0245083. Available from: 10.1371/journal.pone.0245083 PMC7775049 33382862

[67] Galante J, Dufour G, Vainre M, Wagner AP, Stochl J, Benton A, et al. A mindfulness-based intervention to increase resilience to stress in university students (the Mindful Student Study): a pragmatic randomised controlled trial. Lancet Public Heal. 2018;3: e72–e81. Available from: doi: 10.1016/S2468-2667(17)30231-1 29422189 PMC5813792

[68] Chen B, Liu F, Ding S, Ying X, Wang L, Wen Y. Gender differences in factors associated with smartphone addiction: A cross-sectional study among medical college students. BMC Psychiatry. 2017;17: 1–9. Available from: doi: 10.1186/s12888-016-1163-4 29017482 PMC5634822

[69] WHO. Global recommendations on physical activity for health. 2010. Available from: https://www.who.int/dietphysicalactivity/factsheet_recommendations/en/26180873

[70] Ouzzani M, Hammady H, Fedorowicz Z, Elmagarmid A. Rayyan-a web and mobile app for systematic reviews. Syst Rev. 2016;5: 1–10. Available from: doi: 10.1186/s13643-015-0171-7 27919275 PMC5139140

[71] Sallis JF, Prochaska JJ, Taylor WC, Hill JO, Geraci JC. Correlates of physical activity in a national sample of girls and boys in grades 4 through 12. Heal Psychol. 1999;18: 410. Available from: 10.1037//0278-6133.18.4.410 10431943

[72] Guyatt GH, Oxman AD, Vist GE, Kunz R, Falck-Ytter Y, Alonso-Coello P, et al. GRADE: an emerging consensus on rating quality of evidence and strength of recommendations. BMJ. 2008;336: 924–926. Available from: doi: 10.1136/bmj.39489.470347.AD 18436948 PMC2335261

[73] Balshem H, Helfand M, Schünemann HJ, Oxman AD, Kunz R, Brozek J, et al. GRADE guidelines: 3. Rating the quality of evidence. J Clin Epidemiol. 2011;64: 401–406. Available from: doi: 10.1016/j.jclinepi.2010.07.015 21208779

[74] Downs SH, Black N. The feasibility of creating a checklist for the assessment of the methodological quality both of randomised and non-randomised studies of health care interventions. J Epidemiol Community Health. 1998;52: 377–384. Available from: https://jech.bmj.com/content/52/6/377 doi: 10.1136/jech.52.6.377 9764259 PMC1756728

[75] Noll M, de Mendonça CR, de Souza Rosa LP, Silveira EA. Determinants of eating patterns and nutrient intake among adolescent athletes: a systematic review. Nutr J. 2017;16: 46. Available from: doi: 10.1186/s12937-017-0267-0 28754133 PMC5534032

[76] Critical Appraisal Skills Programme. CASP for Systematic Reviews Checklist. Oxford; 2020; 368. Available from: https://casp-uk.net/wp-content/uploads/2018/01/CASP-Systematic-Review-Checklist_2018.pdf%0Ahttps://casp-uk.net/wp-content/uploads/2018/03/CASP-Systematic-Review-Checklist-2018_fillable-form.pdf

[77] Dixon-Woods M, Bonas S, Booth A, Jones DR, Miller T, Sutton AJ, et al. How can systematic reviews incorporate qualitative research? A critical perspective. Qual Res. 2006;6: 27–44. Available from: https://doi.org/10.1177%2F1468794106058867

[78] Noll M, Wedderkopp N, Mendonça CR, Kjaer P. Motor performance and back pain in children and adolescents: A systematic review and meta-analysis protocol. Syst Rev. 2020;9: 4–9. Available from: doi: 10.1186/s13643-019-1261-8 32928303 PMC7491087

[79] Allison KR, Dwyer JJM, Makin S. Perceived barriers to physical activity among high school students. Prev Med. 1999;28: 608–615. Available from: doi: 10.1006/pmed.1999.0489 10404559

[80] Akpınar A. Investigating the barriers preventing adolescents from physical activities in urban green spaces. Urban For Urban Green. 2020;53. Available from: 10.1016/j.ufug.2020.126724

[81] Sherar LB, Gyurcsik NC, Humbert ML, Dyck RF, Fowler-Kerry S, Baxter-Jones ADG. Activity and barriers in girls (8–16 yr) Based on grade and maturity status. Med Sci Sports Exerc. 2009;41: 87–95. Available from: doi: 10.1249/MSS.0b013e31818457e6 19092703

[82] Tappe MK, Duda JL, Ehrnwald PM. Perceived barriers to exercise among adolescents. J Sch Health. 1989;59: 153–155. Available from: doi: 10.1111/j.1746-1561.1989.tb04689.x 2716290

[83] Youssef RM, Al Shafie K, Al-Mukhaini M, Al-Balushi H. Physical activity and perceived barriers among high-school students in Muscat, Oman. East Mediterr Health J. 2013;19: 759–768. Available from: https://pubmed.ncbi.nlm.nih.gov/24313036/ 24313036

[84] Eime RM, Casey MM, Harvey JT, Sawyer NA, Symons CM, Payne WR. Socioecological factors potentially associated with participation in physical activity and sport: A longitudinal study of adolescent girls. J Sci Med Sport. 2015;18: 684–690. Available from: doi: 10.1016/j.jsams.2014.09.012 25308630

[85] Bélanger M, Casey M, Cormier M, Laflamme Filion A, Martin G, Aubut S, et al. Maintenance and decline of physical activity during adolescence: Insights from a qualitative study. Int J Behav Nutr Phys Act. 2011;8. Available from: doi: 10.1186/1479-5868-8-117 22017754 PMC3215642

[86] Satija A, Khandpur N, Satija S, Mathur Gaiha S, Prabhakaran D, Reddy KS, et al. Physical Activity Among Adolescents in India: A Qualitative Study of Barriers and Enablers. Heal Educ Behav Off Publ Soc Public Heal Educ. 2018;45: 926–934. Available from: doi: 10.1177/1090198118778332 29969921

[87] Dambros DD, Lopes LFD, Santos DL. Perceived barriers and physical activity in adolescent students from a Southern Brazilian city. Rev Bras Cineantropometria e Desempenho Hum. 2011;13: 422–428. Available from: 10.1590/1980-0037.2011v13n6p422

[88] Fernandez I, Canet O, Gine-Garriga M. Assessment of physical activity levels, fitness and perceived barriers to physical activity practice in adolescents: cross-sectional study. Eur J Pediatr. 2017;176: 57–65. Available from: doi: 10.1007/s00431-016-2809-4 27858223

[89] Chan JC. Psychological determinants of exercise behavior of nursing students. Contemp Nurse. 2014;49: 60–67. Available from: doi: 10.5172/conu.2014.49.60 25549745

[90] El-Bagoury LS, Hassan AM, AbouSeif HA. Eating attitudes and barriers to healthy eating and physical activity among a sample of university students in Egypt. J Egypt Public Health Assoc. 2017;92: 29–35. Available from: doi: 10.21608/epx.2017.7007 29924925

[91] Gawwad ESA. Stages of change in physical activity, self efficacy and decisional balance among saudi university students. J Family Community Med. 2008;15: 107–115. Available from: https://pubmed.ncbi.nlm.nih.gov/23012176/ 23012176 PMC3377123

[92] Grubbs L, Carter J. The relationship of perceived benefits and barriers to reported exercise behaviors in college undergraduates. Fam Community Health. 2002;25: 76–84. Available from: doi: 10.1097/00003727-200207000-00009 12010117

[93] Gyurcsik NC, Bray SR, Brittain DR. Coping with barriers to vigorous physical activity during transition to university. Fam Community Health. 2004;27: 130–142. Available from: doi: 10.1097/00003727-200404000-00006 15596980

[94] Kgokong D, Parker R. Physical activity in physiotherapy students: Levels of physical activity and perceived benefits and barriers to exercise. South African J Physiother. 2020;76: 1–7. Available from: doi: 10.4102/sajp.v76i1.1399 32391443 PMC7203537

[95] Silliman K, Rodas-Fortier K, Neyman M. A survey of dietary and exercise habits and perceived barriers to following a healthy lifestyle in a college population. Californian J Health Promot. 2004;2: 82–91. Available from: http://citeseerx.ist.psu.edu/viewdoc/download?doi=10.1.1.487.3741&rep=rep1&type=pdf

[96] Vaz M, Bharathi A. An exploratory study of perceptions and practices related to physical activity in women college teachers and students in Bangalore, South India. Health Educ J. 2003;62: 316–325. Available from: https://doi.org/10.1177%2F001789690306200404

[97] Ranasinghe C, Sigera C, Ranasinghe P, Jayawardena R, Ranasinghe ACR, Hills AP, et al. Physical inactivity among physiotherapy undergraduates: Exploring the knowledgepractice gap. BMC Sports Sci Med Rehabil. 2016;8. Available from: doi: 10.1186/s13102-016-0063-8 27980791 PMC5142393

[98] Wattanapisit A, Fungthongcharoen K, Saengow U, Vijitpongjinda S. Physical activity among medical students in Southern Thailand: A mixed methods study. BMJ Open. 2016;6. Available from: doi: 10.1136/bmjopen-2016-013479 27678548 PMC5051498

[99] Padehban V, Negarandeh R, Nikpeyma N. The study of regular physical activity status and perception of barriers for performing it in adolescents. Nurs Pract Today. 2018;5: 347–354. Available from: https://npt.tums.ac.ir/index.php/npt/article/view/384

[100] Robbins LB, Sikorskii A, Hamel LM, Wu T-Y, Wilbur J. Gender comparisons of perceived benefits of and barriers to physical activity in middle school youth. Res Nurs Health. 2009;32: 163–176. Available from: doi: 10.1002/nur.20311 19086055

[101] Serrano JS, Abarca Sos A, Granado JA, Ferrer DC, González LG. Compliance with physical activity guidelines and barriers to physical activity in high school students. Cult Cienc y Deport. 2017;12: 183–194. Available from: https://ccd.ucam.edu/index.php/revista/article/view/946/400

[102] Jodkowska M, Mazur J, Oblacinska A. Perceived barriers to physical activity among Polish adolescents. Przegl Epidemiol. 2015;69: 73–78,169–173. Available from: https://pubmed.ncbi.nlm.nih.gov/25862451/ 25862451

[103] Allison KR, Dwyer JJM, Goldenberg E, Fein A, Yoshida KK, Boutilier M. Male adolescents’ reasons for participating in physical activity, barriers to participation, and suggestions for increasing participation. Adolescence. 2005;40: 155–170. Available from: https://pubmed.ncbi.nlm.nih.gov/15861623/ 15861623

[104] Butt J, Weinberg RS, Breckon JD, Claytor RP. Adolescent physical activity participation and motivational determinants across gender, age, and race. J Phys Act Health. 2011;8: 1074–1083. Available from: https://journals.humankinetics.com/view/journals/jpah/8/8/article-p1074.xml doi: 10.1123/jpah.8.8.1074 22039125

[105] Parobii I, Springer AE, Harrell MB, Gomensoro LM, Fresco MT, Alers N, et al. Exploring physical activity engagement in secondary school students in Montevideo, Uruguay: A qualitative study. Int J Child Adolesc Heal. 2018;11: 47–56. Available from: https://www.ncbi.nlm.nih.gov/pmc/articles/PMC6133320/ 30214660 PMC6133320

[106] Robbins LB, Talley HC, Wu T-Y, Wilbur J. Sixth-grade boys’ perceived benefits of and barriers to physical activity and suggestions for increasing physical activity. J Sch Nurs. 2010;26: 65–77. Available from: doi: 10.1177/1059840509351020 19850952

[107] Sharif Ishak SIZ, Chin YS, Mohd Taib MN, Mohd Shariff Z. Exploration on the Malaysian adolescents’ understanding towards concepts of physical activity, perceived facilitators and barriers in practising an active lifestyle. Br Food J. 2020;122: 3151–3164. Available from: 10.1108/BFJ-01-2020-0049

[108] Zaragoza J, Generelo E, Julian JA, Abarca-Sos A. Barriers to adolescent girls’ participation in physical activity defined by physical activity levels. J Sports Med Phys Fitness. 2011;51: 128–135. Available from: https://pubmed.ncbi.nlm.nih.gov/21297572/ 21297572

[109] Fahlman MM, Hall HL, Lock R. Ethnic and socioeconomic comparisons of fitness, activity levels, and barriers to exercise in high school females. J Sch Health. 2006;76: 12–17. Available from: doi: 10.1111/j.1746-1561.2006.00061.x 16457680

[110] Robbins LB, Pender NJ, Kazanis AS. Barriers to physical activity perceived by adolescent girls. J Midwifery Women’s Heal. 2003;48: 206–212. Available from: doi: 10.1016/s1526-9523(03)00054-0 12764306

[111] Dwyer JJM, Allison KR, Goldenberg ER, Fein AJ, Yoshida KK, Boutilier MA. Adolescent girls’ perceived barriers to participation in physical activity. Adolescence. 2006;41: 75–89. Available from: https://pubmed.ncbi.nlm.nih.gov/16689442/ 16689442

[112] Wetton AR, Radley R, Jones AR, Pearce MS. What are the barriers which discourage 15–16 year-old girls from participating in team sports and how can we overcome them? Biomed Res Int. 2013;2013. Available from: 10.1155/2013/738705 PMC3773401 24073416

[113] Abdelghaffar E-A, Hicham EK, Siham B, Samira EF, Youness EA. Perspectives of adolescents, parents, and teachers on barriers and facilitators of physical activity among school-age adolescents: A qualitative analysis. Environ Health Prev Med. 2019;24. Available from: 10.1186/s12199-019-0775-y PMC6454728 30961543

[114] Hohepa M, Schofield G, Kolt GS. Physical activity: what do high school students think? J Adolesc Heal. 2006;39: 328–336. Available from: doi: 10.1016/j.jadohealth.2005.12.024 16919793

[115] Moore JB, Jilcott SB, Shores KA, Evenson KR, Brownson RC, Novick LF. A qualitative examination of perceived barriers and facilitators of physical activity for urban and rural youth. Health Educ Res. 2010;25: 355–367. Available from: doi: 10.1093/her/cyq004 20167607 PMC10170971

[116] Hsu Y-W, Chou C-P, Nguyen-Rodriguez ST, McClain AD, Belcher BR, Spruijt-Metz D. Influences of social support, perceived barriers, and negative meanings of physical activity on physical activity in middle school students. J Phys Act Health. 2011;8: 210–219. Available from: doi: 10.1123/jpah.8.2.210 21415448 PMC8098645

[117] Gunnell KE, Brunet J, Wing EK, Bélanger M. Measuring Perceived Barriers to Physical Activity in Adolescents. Pediatr Exerc Sci. 2015;27: 252–261. Available from: doi: 10.1123/pes.2014-0067 25679535

[118] Kulavic K, Hultquist CN, McLester JR. A comparison of motivational factors and barriers to physical activity among traditional versus nontraditional college students. J Am Coll Health. 2013;61: 60–66. Available from: doi: 10.1080/07448481.2012.753890 23409855

[119] Sousa TF de, Fonseca SA, Barbosa AR. Perceived barriers by university students in relation the leisure-time physical activity. Brazilian J Kineanthropometry Hum Perform. 2013;15: 164–173. Available from: 10.1590/1980-0037.2013v15n2p164

[120] Frederick GM, Williams ER, Castillo-Hernández IM, Evans EM. Physical activity and perceived benefits, but not barriers, to exercise differ by sex and school year among college students. J Am Coll Heal. 2020;0: 1–8. Available from: 10.1080/07448481.2020.1800711 32813632

[121] Samara A, Nistrup A, Al-Rammah TY, Aro AR. Lack of facilities rather than sociocultural factors as the primary barrier to physical activity among female Saudi university students. Int J Womens Health. 2015;7: 279–286. Available from: doi: 10.2147/IJWH.S80680 25834468 PMC4358666

[122] Burton NW, Barber BL, Khan A. A Qualitative Study of Barriers and Enablers of Physical Activity among Female Emirati University Students. Int J Environ Res Public Health. 2021;18: 3380. Available from: doi: 10.3390/ijerph18073380 33805174 PMC8037841

[123] Laar RA, Shi S, Ashraf MA. Participation of pakistani female students in physical activities: Religious, cultural, and socioeconomic factors. Religions. 2019;10. Available from: 10.3390/rel10110617

[124] Nishimwe-Niyimbanira R, Muzindutsi PF. Antecedents of participation in physical activity among generation Y at a South African higher education institution. Mediterr J Soc Sci. 2014;5: 290–298. Available from: 10.5901/mjss.2014.v5n21p291

[125] Sukys S, Cesnaitiene VJ, Emeljanovas A, Mieziene B, Valantine I, Ossowski ZM. Reasons and Barriers for University Students’ Leisure-Time Physical Activity: Moderating Effect of Health Education. Percept Mot Skills. 2019;126: 1084–1100. Available from: doi: 10.1177/0031512519869089 31407961

[126] Anjali, Sabharwal M. Perceived barriers of young adults for participation in physical activity. Curr Res Nutr Food Sci. 2018;6: 437–449. Available from: 10.12944/CRNFSJ.6.2.18

[127] Awadalla NJ, Aboelyazed AE, Hassanein MA, Khalil SN, Aftab R, Gaballa II, et al. Assessment of physical inactivity and perceived barriers to physical activity among health college students, south-western Saudi Arabia. East Mediterr Health J. 2014;20: 596–604. Available from: https://pubmed.ncbi.nlm.nih.gov/25356690/ 25356690

[128] El-Gilany AH, Badawi K, El-Khawaga G, Awadalla N. Physical activity profile of students in Mansoura University, Egypt. East Mediterr Health J. 2011;17: 694–702. Available from: https://pubmed.ncbi.nlm.nih.gov/21977573/ 21977573

[129] Pandolfo KCM, Minuzzi T, Machado RR, Lopes LFD, Azambuja CR, Santos DL dos. Perceived barriers to physical activity practice in high school students. Brazilian J Kinanthropometry Hum Perform. 2016;18: 567. Available from: 10.1590/1980-0037.2016v18n5p567

[130] Rosselli M, Ermini E, Tosi B, Boddi M, Stefani L, Toncelli L, et al. Gender differences in barriers to physical activity among adolescents. Nutr Metab Cardiovasc Dis. 2020;30: 1582–1589. Available from: doi: 10.1016/j.numecd.2020.05.005 32605880

[131] De Camargo EM, López-Gil JF, De Campos W. Comparison of perceived barriers to physical activity according to sex and physical activity level. Cuad Psicol del Deport. 2021;21: 204–215. Available from: 10.6018/cpd.371571

[132] Musaiger AO, Al-Mannai M, Tayyem R, Al-Lalla O, Ali EYA, Kalam F, et al. Perceived barriers to healthy eating and physical activity among adolescents in seven arab countries: A cross-cultural study. Sci World J. 2013;2013. Available from: doi: 10.1155/2013/232164 24348144 PMC3848306

[133] Portela-pino I, Antonio L. Gender Diffrences in Motivation and Barriers for The Practice of Physical Exercise in Adolescence. 2019. Available from: 10.3390/ijerph17010168 PMC6981955 31881707

[134] Santos MS, Hino AAF, Reis RS, Rodriguez-Añez CR. Prevalence of barriers for physical activity in adolescents. Rev Bras Epidemiol. 2010;13: 94–104. Available from: doi: 10.1590/s1415-790x2010000100009 20683558

[135] Dias DF, Loch MR, Ronque ER V. Perceived barriers to leisure-time physical activity and associated factors in adolescents. Cienc e Saude Coletiva. 2015;20: 3339–3350. Available from: doi: 10.1590/1413-812320152011.00592014 26602712

[136] Ramírez-Vélez R, Tordecilla-Sanders A, Laverde D, Hernández-Novoa JG, Ríos M, Rubio F, et al. The prevalence of barriers for Colombian college students engaging in physical activity. Nutr Hosp. 2015;31: 858–865. Available from: http://www.aulamedica.es/nh/pdf/7737.pdf 10.3305/nh.2015.31.2.7737 25617574

[137] Public Health Service. Department of Health and Human Services.(1999). Promoting physical activity: a guide for community action. Human Kinetics.

[138] Garcia LMT, Fisberg M. Physical activities and barriers reported by adolescents attending a health service. Rev Bras Cineantropometria e Desempenho Hum. 2011;13: 163–169. Available from: 10.5007/1980-0037.2011v13n3p163

[139] Santos MS, Fermino RC, Reis RS, Cassou AC, Añez CRR. Barriers related to physical activity practice in adolescents. A focus-group study. Rev Bras Cineantropometria e Desempenho Hum. 2010;12: 137–143. Available from: https://portalrevistas.ucb.br/index.php/RBCM/article/viewFile/727/730

[140] Blake H, Stanulewicz N, Mcgill F. Predictors of physical activity and barriers to exercise in nursing and medical students. J Adv Nurs. 2017;73: 917–929. Available from: doi: 10.1111/jan.13181 27731886

[141] Vieira VR, Da Silva JVP. Barriers to the practice of physical activities in the leisure of Brazilians: systematic review. Pensar a Prática. 2019;22: 1–22. Available from: 10.5216/rpp.v22.54448

[142] Young DR, Felton GM, Grieser M, Elder JP, Johnson C, Lee JS, et al. Policies and opportunities for physical activity in middle school environments. J Sch Health. 2007;77: 41–47. Available from: doi: 10.1111/j.1746-1561.2007.00161.x 17212759 PMC2475674

[143] Hilger-Kolb J, Loerbroks A, Diehl K. “When I have time pressure, sport is the first thing that is cancelled”: A mixed-methods study on barriers to physical activity among university students in Germany. J Sports Sci. 2020;38: 2479–2488. Available from: doi: 10.1080/02640414.2020.1792159 32658595

[144] Deliens T, Deforche B, De Bourdeaudhuij I, Clarys P. Determinants of physical activity and sedentary behaviour in university students: A qualitative study using focus group discussions. BMC Public Health. 2015;15: 1–9. Available from: doi: 10.1186/1471-2458-15-1 25881120 PMC4349731

[145] Chaabane S, Chaabna K, Doraiswamy S, Mamtani R, Cheema S. Barriers and Facilitators Associated with Physical Activity in the Middle East and North Africa Region: A Systematic Overview. International Journal of Environmental Research and Public Health. 2021. Available from: doi: 10.3390/ijerph18041647 33572229 PMC7914747

[146] Harrison AL, Taylor NF, Shields N, Frawley HC. Attitudes, barriers and enablers to physical activity in pregnant women: a systematic review. J Physiother. 2018;64: 24–32. Available from: doi: 10.1016/j.jphys.2017.11.012 29289592

[147] Supples MW, Rivard MK, Cash RE, Chrzan K, Panchal AR, McGinnis HD. Barriers to Physical Activity Among Emergency Medical Services Professionals. J Phys Act Heal. 18: 304–309. Available from: doi: 10.1123/jpah.2020-0305 33567402

[148] Sharara E, Akik C, Ghattas H, Makhlouf Obermeyer C. Physical inactivity, gender and culture in Arab countries: A systematic assessment of the literature. BMC Public Health. 2018;18: 1–19. Available from: doi: 10.1186/s12889-018-5472-z 29776343 PMC5960209

[149] Pedersen MR, Hansen AF, Elmose-Østerlund K. Motives and Barriers Related to Physical Activity and Sport across Social Backgrounds: Implications for Health Promotion. International Journal of Environmental Research and Public Health. 2021. Available from: doi: 10.3390/ijerph18115810 34071630 PMC8198157

[150] Brand R, Cheval B. Theories to Explain Exercise Motivation and Physical Inactivity: Ways of Expanding Our Current Theoretical Perspective. Front Psychol. 2019;10: 1147. Available from: doi: 10.3389/fpsyg.2019.01147 31164856 PMC6536603

[151] Rhodes RE, McEwan D, Rebar AL. Theories of physical activity behaviour change: A history and synthesis of approaches. Psychol Sport Exerc. 2019;42: 100–109. Available from: 10.1016/j.psychsport.2018.11.010

[152] Buchan DS, Ollis S, Thomas NE, Baker JS. Physical Activity Behaviour: An Overview of Current and Emergent Theoretical Practices. Gorin AA, editor. J Obes. 2012;2012: 546459. Available from: doi: 10.1155/2012/546459 22778918 PMC3388376

[153] Bandura A. National Inst of Mental Health. (1986). Social foundations of thought and action: A social cognitive theory. Prentice-Hall, Inc.

[154] Rosenstock I, Strecher V, Becker MH. Social Learning Theory and the Health Belief Model. Heal Educ Behav. 1988;15: 175–183. Available from: doi: 10.1177/109019818801500203 3378902

[155] Ajzen I. The theory of planned behavior. Organ Behav Hum Decis Process. 1991;50: 179–211. Available from: 10.1016/0749-5978(91)90020-T

[156] Prochaska J, Diclemente C. Stages and processes of self-change of smoking: toward an integrative model of change. J Consult Clin Psychol. 1983;51 3: 390–395. Available from: doi: 10.1037//0022-006x.51.3.390 6863699

[157] Schwarzer R. Modeling health behavior change: how to predict and modify the adoption and maintenance of health behaviors. Appl Psychol An Int Rev. 2008;57: 1–29. Available from: 10.1111/j.1464-0597.2007.00325.x

[158] Humpel N, Owen N, Leslie E. Environmental factors associated with adults’ participation in physical activity: a review. Am J Prev Med. 2002;22: 188–199. Available from: doi: 10.1016/s0749-3797(01)00426-3 11897464

[159] Stokols D. Social ecology and behavioral medicine: implications for training, practice, and policy. Behav Med. 2000;26: 129–138. Available from: doi: 10.1080/08964280009595760 11209593

[160] Guldager JD, Andersen PT, von Seelen J, Leppin A. Physical activity school intervention: context matters. Health Educ Res. 2018;33: 232–242. Available from: doi: 10.1093/her/cyy012 29741620

[161] Vaquero-Solís M, Gallego DI, Tapia-Serrano MÁ, Pulido JJ, Sánchez-Miguel PA. School-based Physical Activity Interventions in Children and Adolescents: A Systematic Review. Int J Environ Res Public Health. 2020;17: 999. Available from: doi: 10.3390/ijerph17030999 32033392 PMC7037705

[162] Abu-Omar K, Rütten A, Burlacu I, Schätzlein V, Messing S, Suhrcke M. The cost-effectiveness of physical activity interventions: A systematic review of reviews. Prev Med Reports. 2017;8: 72–78. Available from: 10.1016/j.pmedr.2017.08.006 PMC5573782 28856084

[163] Schlund A, Reimers AK, Bucksch J, Brindley C, Schulze C, Puil L, et al. Do Intervention Studies to Promote Physical Activity and Reduce Sedentary Behavior in Children and Adolescents Take Sex/Gender Into Account? A Systematic Review. J Phys Act Heal. 2021;18: 461–468. Available from: doi: 10.1123/jpah.2020-0666 33668018

[164] Kalajas-Tilga H, Koka A, Hein V, Tilga H, Raudsepp L. Motivational processes in physical education and objectively measured physical activity among adolescents. J Sport Heal Sci. 2020;9: 462–471. Available from: doi: 10.1016/j.jshs.2019.06.001 32928449 PMC7498624

[165] Memon AR, Ali B, Memon AUR, Ahmed I, Feroz J. Motivation and factors affecting sports participation: a cross-sectional study on female medical students in Pakistan. J Pak Med Assoc. 2018;68: 1327–1333. Available from: https://jpma.org.pk/article-details/8846?article_id=8846 30317259

[166] Welk GJ, Kim Y. Context of Physical Activity in a Representative Sample of Adults. Med Sci Sports Exerc. 2015;47: 2102–2110. Available from: doi: 10.1249/MSS.0000000000000641 25699482 PMC4544620

[167] Abbasi IN. Socio-cultural Barriers to Attaining Recommended Levels of Physical Activity among Females: A Review of Literature. Quest. 2014;66: 448–467. Available from: 10.1080/00336297.2014.955118

[168] Burke SM, Carron A V, Eys MA. Physical activity context: Preferences of university students. Psychol Sport Exerc. 2006;7: 1–13. Available from: 10.1016/j.psychsport.2005.03.002

[169] Kuo J, Schmitz KH, Evenson KR, McKenzie TL, Jobe JB, Rung AL, et al. Physical and social contexts of physical activities among adolescent girls. J Phys Act Health. 2009;6: 144–152. Available from: doi: 10.1123/jpah.6.2.144 19420391 PMC4959469

[170] Aljayyousi GF, Abu Munshar M, Al-Salim F, Osman ER. Addressing context to understand physical activity among Muslim university students: the role of gender, family, and culture. BMC Public Health. 2019;19: 1452. Available from: doi: 10.1186/s12889-019-7670-8 31690307 PMC6829810

[171] Lizandra J, Devís-Devís J, Valencia-Peris A, Tomás JM, Peiró-Velert C. Screen time and moderate-to-vigorous physical activity changes and displacement in adolescence: A prospective cohort study. Eur J Sport Sci. 2019;19: 686–695. Available from: doi: 10.1080/17461391.2018.1548649 30550370

[172] Pearson N, Sherar LB, Hamer M. Prevalence and Correlates of Meeting Sleep, Screen-Time, and Physical Activity Guidelines Among Adolescents in the United Kingdom. JAMA Pediatr. 2019;173: 993–994. Available from: http://jamanetwork.com/article.aspx?doi=10.1001/jamapediatrics.2019.2822 31449287 10.1001/jamapediatrics.2019.2822 PMC6714019

