# Supplementary material for: Barriers to high school and university students’ physical activity: A systematic review
Source: PLoS One. 2022 Apr 4;17(4):e0265913. doi: 10.1371/journal.pone.0265913 (PMC8979430; doi:10.1371/journal.pone.0265913)
Supplement: S1 Checklist — (DOCX) [file pone.0265913.s001.docx]

| **Supplementary Table 1.** Search strategy performed November 05, 2021. | |
| --- | --- |
| **CINAHL** | ( TITLE-ABS-KEY ( "physical activity" OR "sedentary lifestyle" OR "physical inactivity" OR "sedentary behavior" ) ) AND ( TITLE-ABS-KEY ( "adolescence" OR "adolescent" OR "college students" OR "high school student" OR "student" OR "teen" OR "teenagers" OR "undergraduate students" OR "university students" OR " youth" ) ) AND ( TITLE-ABS-KEY ( "barriers" OR "challenges" OR "difficulties" OR "obstacles" ) ) |
| **Cochrane Library** | "physical activity" OR "sedentary lifestyle" OR " physical inactivity" OR "sedentary behavior" in Title Abstract Keyword AND "barriers" OR "challenges" OR "difficulties" OR "obstacles" in Title Abstract Keyword AND "adolescent" OR "student" OR "teen" OR "youth" in Title Abstract Keyword |
| **Embase** | ( TITLE-ABS-KEY ( "physical activity" OR "sedentary lifestyle" OR "physical inactivity" OR "sedentary behavior" ) ) AND ( TITLE-ABS-KEY ( "adolescence" OR "adolescent" OR "college students" OR "high school student" OR "student" OR "teen" OR "teenagers" OR "undergraduate students" OR "university students" OR " youth" ) ) AND ( TITLE-ABS-KEY ( "barriers" OR "challenges" OR "difficulties" OR "obstacles" ) ) |
| **PubMed** | ((("exercise"[MeSH Terms] AND "journal article"[Publication Type]) OR ("physical inactivity"[Title/Abstract] AND "journal article"[Publication Type]) OR ("sedentary behavior"[MeSH Terms] AND "journal article"[Publication Type]) OR ("sedentary behavior"[Title/Abstract] AND "journal article"[Publication Type])) AND "journal article"[Publication Type] AND ((("adolescent"[MeSH Terms] AND "journal article"[Publication Type]) OR ("college students"[Title/Abstract] AND "journal article"[Publication Type]) OR ("students"[MeSH Terms] AND "journal article"[Publication Type]) OR ("adolescent"[MeSH Terms] AND "journal article"[Publication Type]) OR ("high school students"[Title/Abstract] AND "journal article"[Publication Type]) OR ("undergraduate students"[Title/Abstract] AND "journal article"[Publication Type]) OR 12[UID]) AND "journal article"[Publication Type]) AND ((("barriers"[Title/Abstract] AND "journal article"[Publication Type]) OR ("difficulties"[Title/Abstract] AND "journal article"[Publication Type]) OR ("obstacles"[Title/Abstract] AND "journal article"[Publication Type]) OR ("challenges"[Title/Abstract] AND "journal article"[Publication Type])) AND "journal article"[Publication Type])) AND ((journalarticle[Filter]) |
| **Scopus** | ( TITLE-ABS-KEY ( "physical activity" OR "sedentary lifestyle" OR "physical inactivity" OR "sedentary behavior" ) ) AND ( TITLE-ABS-KEY ( "adolescence" OR "adolescent" OR "college students" OR "high school student" OR "student" OR "teen" OR "teenagers" OR "undergraduate students" OR "university students" OR " youth") ) AND ( TITLE-ABS-KEY ( "barriers" OR "challenges" OR "difficulties" OR "obstacles" ) ) |
