# Supplementary material for: Barriers to high school and university students’ physical activity: A systematic review
Source: PLoS One. 2022 Apr 4;17(4):e0265913. doi: 10.1371/journal.pone.0265913 (PMC8979430; doi:10.1371/journal.pone.0265913)
Supplement: S1 Table — (DOCX) [file pone.0265913.s002.docx]

| **Section and Topic** | **Item #** | **Checklist item** | **Location where item is reported** |
| --- | --- | --- | --- |
| **TITLE** | | |  |
| Title | 1 | Barriers to high school and university students’ physical activity: a systematic review | 1 |
| **ABSTRACT** | | |  |
| Abstract | 2 | Physical inactivity commonly occurs throughout one’s life, particularly during adolescence and young adulthood. Multiple factors can negatively influence participation in physical activity, but there has been no review examining the barriers to physical activity among high school and university students. Therefore, the aim of this systematic review was to summarize evidence of barriers to the practice of physical activity among high school and university students. The literature search was conducted without time limits using five databases, including CINAHL, Cochrane Library, Embase, PubMed, and Scopus. In total, 59 studies (37 with high school students [n=22,908] and 22 with university students [n=15,411]) were included. The main barriers identified in high school and university students were lack of time, lack of motivation, and lack of accessible places. These findings may be useful in designing and implementing evidence-informed interventions and programs for physical activity promotion in students. | 1 |
| **INTRODUCTION** | | |  |
| Rationale | 3 | As far as we know, only one systematic review from 2014 [56] and an updated systematic review [57] have been published on barriers to physical activity in adolescents. However, these reviews are limited to only studies covering a specific age group (adolescents between 13 and 18 years old) [56,57], which excludes undergraduate university students. Therefore, there is a need for further research focusing on diverse populations (e.g., children, adolescents, university students) and study designs to advance the knowledge in this area [57,58]. | 3 |
| Objectives | 4 | Thus, this systematic review aimed to identify barriers to the practice of physical activity among high school, college, and university students. | 4 |
| **METHODS** | | |  |
| Eligibility criteria | 5 | The eligibility criteria were specified according to the Population, Exposure, Comparator, and Outcomes (PEOS) framework for the research question. In which "P" represented high school and/or university students, comprising adolescents or adults of both sexes aged between 10–30 years; "E" represented barriers to physical activity; “O" represented the practice of physical activity. "S" represented studies with qualitative and quantitative designs published during any year in peer-reviewed journals in English language. The World Health Organization defines ‘adolescents’ as individuals in the 10–19 years age group and ‘youth’ as individuals in the 15–24 year age group; thus, ‘young people’ are individuals in the age range from 10 to 24 years [54]. We had the age group between 10 and 30 years. The extension of age range to 30 years was justified by the fact that this age range would also cover university students who are enrolled in postgraduate courses [55–57]. | 5 |
| Information sources | 6 | CINAHL, Cochrane Library, Embase, PubMed and Scopus. | 4 |
| Search strategy | 7 | 1 = “physical activity” OR “sedentary lifestyle” OR “physical inactivity” OR “sedentary behavior”  2 = “adolescence” OR “adolescent" OR “college students” OR “high school student” OR “student” OR “teen” OR “teenagers” OR “undergraduate student” OR “university student” OR “youth”  3 = “barriers” OR “challenges” OR “difficulties” OR “obstacles”  4 = 1 AND 2 AND 3 | Suppplementary Table 1 |
| Selection process | 8 | All studies with qualitative and quantitative designs published during any year in peer-reviewed journals in English, Spanish, or Portuguese. | 5 |
| Data collection process | 9 | First stage of reading titles and abstracts, second stage full reading of the article. | 6 |
| Data items | 10a | Following data were extracted from the included studies: author and year of the publication, type of study, country of origin, population, sex, age group of the population, instrument used for data collection and the barriers to physical activity. We categorized the results into two groups: (a) high school students and (b) university students. | 6 |
|  | 10b |  |  |
| Study risk of bias assessment | 11 | After the full-text screening is completed, the articles included will be assessed for quality using the recommendations (GRADE) and Critical Appraisal Skills Program Qualitative Research Checklist (CASP). The risk of bias will be assessed independently by two reviewers. | 6 |
| Effect measures | 12 | Not applied. | - |
| Synthesis methods | 13a | The information was extracted independently by two reviewers (R.M.F.S. and C.R.M.), and the disagreements were resolved by a third reviewer (M.N.). | 6 |
|  | 13b | Describe any methods required to prepare the data for presentation or synthesis, such as handling of missing summary statistics, or data conversions. |  |
|  | 13c | Table 1, 2, 3 and 4. | 10-14 |
|  | 13d | Not applied. | - |
|  | 13e | Not applied. | - |
|  | 13f | Not applied. | - |
| Reporting bias assessment | 14 | After the full-text screening is completed, the articles included will be assessed for quality using the recommendations (GRADE) and Critical Appraisal Skills Program Qualitative Research Checklist (CASP). The risk of bias will be assessed independently by two reviewers. | 7 |
| Certainty assessment | 15 | Not applied. | - |
| **RESULTS** | | |  |
| Study selection | 16a | Preferred Reporting Items for Systematic Reviews and Meta-Analyses flow diagram for study selection. | Figure 1 |
|  | 16b | The eligibility criteria were specified according to the Population, Exposure, Comparator, and Outcomes (PEOS) framework for the research question. In which "P" represented high school and/or university students, comprising adolescents or adults of both sexes aged between 10–30 years; "E" represented barriers to physical activity; “O" represented the practice of physical activity. "S" represented studies with qualitative and quantitative designs published during any year in peer-reviewed journals in English language.The World Health Organization defines ‘adolescents’ as individuals in the 10–19 years age group and ‘youth’ as individuals in the 15–24 year age group; thus, ‘young people’ are individuals in the age range from 10 to 24 years [54]. We had the age group between 10 and 30 years. The extension of age range to 30 years was justified by the fact that this age range would also cover university students who are enrolled in postgraduate courses [55–57]. | 5 |
| Study characteristics | 17 | Table 1, 2, 3 and 4. | 10-14 |
| Risk of bias in studies | 18 | Table 5 and Table 6. | 16-18 |
| Results of individual studies | 19 | Table 1, 2, 3 and 4. | 10-14 |
| Results of syntheses | 20a | Tables: 1,2, 3, 4, 5 and 6. | 10-18 |
|  | 20b | Not applied. | - |
|  | 20c | Not applied. | - |
|  | 20d | Not applied. | - |
| Reporting biases | 21 | Not applied. | - |
| Certainty of evidence | 22 | Not applied. | - |
| **DISCUSSION** | | |  |
| Discussion | 23a | Although this review, to the best possible we know, is the first to examine barriers to physical activity among high school and university students, some limitations of this review should be acknowledged. For example, the restriction for studies published in English only might have led to exclusion of potential studies published in other languages. The studies included in this review provide low quality of evidence and had a great diversity of instruments for the identification of barriers to physical activity in students. Therefore, future studies should have strong methodological rigor to generate better evidence, and also the need to be based on the context-sensitive, standardized global instrument to determine barriers to physical activity among high school and university students. The use of standardized global instruments for physical activity among university students has also been advocated in a recent review [35]. | 19 |
|  | 23b |  |  |
|  | 23c |  |  |
|  | 23d |  |  |
| **OTHER INFORMATION** | | |  |
| Registration and protocol | 24a | The protocol for this review was recently published [53] and it was registered in the PROSPERO database (CRD42020198899). | 5 |
|  | 24b |  |  |
|  | 24c |  |  |
| Support | 25 | This research did not receive any specific grant from funding agencies in the public, commercial, or not-for-profit sectors.  We thank the Federal Institute Goiano and the Child and Adolescent Health Research Group (GPSaCA - [www.gpsaca.com.br](file:///C:\Users\Leandro\Desktop\GPSaCA\Artigo%20da%20Revisão%20Sistemática\www.gpsaca.com.br)) for their support. | 21 |
| Competing interests | 26 | The authors report no declarations of interest. | 21 |
| Availability of data, code and other materials | 27 | Not applied . | - |

*From:*  Page MJ, McKenzie JE, Bossuyt PM, Boutron I, Hoffmann TC, Mulrow CD, et al. The PRISMA 2020 statement: an updated guideline for reporting systematic reviews. BMJ 2021;372:n71. doi: 10.1136/bmj.n71

For more information, visit: <http://www.prisma-statement.org/>
